# Does the Intra‐Atomic Deformation Energy of Interacting Quantum Atoms Represent Steric Energy?

**DOI:** 10.1002/open.201800275

**Published:** 2019-02-08

**Authors:** Benjamin C. B. Symons, Dominic J. Williamson, Campbell M. Brooks, Alex L. Wilson, Paul L. A. Popelier

**Affiliations:** ^1^ Manchester Institute of Biotechnology (MIB) 131 Princess Street Manchester M1 7DN Great Britain; ^2^ School of Chemistry University of Manchester Oxford Road Manchester M13 9PL Great Britain

**Keywords:** quantum chemical topology (QCT), steric effects, van der Waals complexes, interacting quantum atoms (IQA), short-range repulsion potential

## Abstract

We show that the mutual, through‐space compression of atomic volume experienced by approaching topological atoms causes an exponential increase in the intra‐atomic energy of those atoms, regardless of approach orientation. This insight was obtained using the modern energy partitioning method called interacting quantum atoms (IQA). This behaviour is consistent for all atoms except hydrogen, which can behave differently depending on its environment. Whilst all atoms experience charge transfer when they interact, the intra‐atomic energy of the hydrogen atom is more vulnerable to these changes than larger atoms. The difference in behaviour is found to be due to hydrogen's lack of a core of electrons, which, in heavier atoms, consistently provide repulsion when compressed. As such, hydrogen atoms do not always provide steric hindrance. In accounting for hydrogen's unusual behaviour and demonstrating the exponential character of the intra‐atomic energy in all other atoms, we provide evidence for IQA's intra‐atomic energy as a quantitative description of steric energy.

## Graphical abstract

**Energy boost**: The mutual, through‐space compression of atomic volume experienced by approaching topological atoms is demonstrated to cause an exponential increase in the intra‐atomic energy of those atoms, regardless of approach orientation. This insight is obtained using the modern energy partitioning method called interacting quantum atoms (IQA). This behaviour is consistent for all atoms except hydrogen, which can behave differently depending on its environment. Evidence is provided for IQA's intra‐atomic energy as a quantitative description of steric energy.

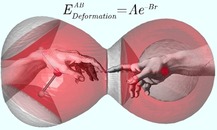

## 1 Introduction

Various chemical concepts predate quantum mechanics and thus quantum chemistry. To name a couple of examples, this is the case for the concept of the chemical bond and also for that of short‐range repulsion, the subject of this paper. In the days of Slater's PhD research on solid state compressibility under high pressure, the nature of short‐range repulsion was still a mystery. Later in his career, Slater set out to unravel this mystery using quantum mechanics. Similarly, the covalent bond, an idea traceable to Lewis already in 1916, was only later (1927) connected to quantum mechanics by Heitler and London. Linking chemical concepts and chemical intuition to quantum mechanics is a valuable and necessary activity for the general “health” of Chemistry as a science. However, it appears that the great numerical success of quantum chemistry made it focus much more on accurate property prediction rather than enhancing or even correcting chemical insight. Furthermore, the mathematical success of the popular molecular orbital *ansatz* has not helped in bridging the gap between chemical insight and modern wavefunctions. Hence, showing how chemical insight emerges from an underlying quantum reality remains an active area of research.

In this work we focus on short‐range repulsion, which can be associated with steric effects. Although steric effects are chemically plausible, and even borrow from daily life experience, they must ultimately be traced back to energy effects. Classical repulsive potentials, such as the r^−12^ part of the Lennard‐Jones potential, have been known for a long time. In fact, a more general repulsive energy term (A/R^n^) was first proposed by Mie already in 1906. Lennard‐Jones then adopted Mie's potential in the early 1920s, still before the birth of quantum mechanics. Soon after, Born and Mayer suggested^1^ that interatomic repulsion should have an approximate exponential dependence on internuclear distance. Later, in 1938, Buckingham proposed^2^ his exponential potential as a simplification to the Lennard‐Jones potential in a theoretical study of the equation of state of gaseous helium, neon and argon. However, in spite of its deficiencies the Lennard‐Jones potential enjoyed an immediate popularity over the Buckingham potential due to the cost of evaluating exponentials functions in the days of early computers.^3^

The current work builds on previous work,^4^ which for the first time established a quantitative link between the internal energy of topological atoms^5^ and classical interatomic repulsive potentials. Topological atoms^6^ are quantum atoms featuring in an increasingly popular energy decomposition scheme called Interacting Quantum Atoms (IQA).^7^ IQA's growing use is demonstrated by its wide variety of applications^8^, ^9^, ^10^, ^11^, ^12^, ^13^, ^14^, ^15^ ranging from halogen bond formation^16^ to substituent effects in electronically excited states,^17^ just to name a few. The IQA partitioning scheme is an attractive candidate to serve as a bridge between chemical insight and present‐day wavefunctions. A combination of IQA and the newly proposed Relative Energy Gradient (REG) method^18^ has delivered crisp chemical insight explaining the chemical nature of a traditional hydrogen bond,^18^ enzymatic hydrolysis,^19^ the fluorine gauche effect^20^ and the origin of rotation barriers in biphenyl.^21^ IQA provides four types of energy, which are all well‐defined at atomistic level: intra‐atomic energy (which we showed^4^ corresponds to sterics), electrostatic energy (with a link to multipole moments^22^), exchange energy (related to bond order^23^) and correlation energy (expanding dispersion^24^). Traditional (and older) energy partitioning schemes come with a number of typical problems as recently reviewed.^25^ For example, at close intermolecular distances and with large basis sets, the separation of charge transfer and polarization becomes increasingly ill‐defined, and numerical instabilities may occur. The space‐filling nature^26^ of the topological atoms, which is at the heart of IQA, makes sure that IQA does not suffer from this difficulty. A second example of a snag in a traditional energy decomposition scheme is the interpretation of the so‐called deformation energy term in NEDA (Natural Energy Decomposition Analysis),^27^ which problematically includes both the contribution of Pauli repulsion as well as the intra‐atomic (or “self”) energy penalty. This issue is closer to the core of the current work but again IQA does not^28^ suffer from this drawback. A current concern arisen^29^ in connection with an IQA study on perfluorinated hydrocarbons was soon met.^30^ We expect that this particular case study will benefit from a REG analysis, which copes well with competing IQA contributions.

It is with this IQA background in mind that the scene for the current contribution can be set more precisely. The previous contribution,^4^ which we henceforth refer to as Paper I, corroborated that the sum of the deformation energy of atom *A* and atom *B* is effectively the short‐range repulsion energy between two atoms. The deformation energy of a given atom is the intra‐atomic energy of that atom within the molecular system (or van der Waals complex) minus the intra‐atomic energy of that atom within the isolated (or “free”) monomer. Paper I showed that this deformation energy is better fitted by a Buckingham‐type potential than by the Lennard‐Jones r^−12^ repulsive potential. This is good news because the former is considered to be more accurate and realistic. Secondly, Paper I also showed that topological atoms “feel” each other's presence over longer distances than expected. Thirdly, that work also established that so‐called mixing rules exist within operational energy error bars. In all of Paper I the monomers constituting the investigated van der Waals complexes approached each other along the line linking the two non‐hydrogen atoms. Although the work of Paper I is valuable by itself and ground‐breaking as a proof‐of‐concept, the question arises of its general validity. Is the mapping of sterics and short‐range interatomic repulsion to topological deformation energy still valid for less artificial approaches? Furthermore, is the established understanding still valid when hydrogen atoms are involved in the path of approach? This work will answer those questions and provide a deeper understanding of the nature of the topological intra‐atomic energy.

Finally we mention that the potentials proposed here are possibly useful in the development of the novel force field FFLUX,^31^,^32^ which uses machine learning (i. e. kriging) to predict the multipole moments^33^,^34^ and energies of topological atoms. However, their use in the simulation of liquid water, for example, would be transitional because FFLUX's ultimate goal is to calculate and express all energies by kriged potentials, and also to avoid (distributed) polarisabilities^35^.

## 2 Background and Methodology

### 2.1 Interacting Quantum Atoms (IQA)

The Interacting Quantum Atoms (IQA) formalism^7^ is an energy partitioning scheme based on the Quantum Theory of Atoms in Molecules (QTAIM)^5^ wherein atoms are defined as space‐filling parameter‐free volumes. Both IQA and QTAIM are part of an overarching approach called Quantum Chemical Topology (QCT),^36^ a name first coined^37^ in 2003, and the Latin American contributions to which were very recently reviewed.^38^ In the IQA formalism, the total energy of a system is defined as the sum of the intra‐atomic energies, denoted $E_{\mathrm{intra}}^{A}$ , and the inter‐atomic energies $E_{\mathrm{inter}}^{AB}$ . Note that no assumptions are made about whether atoms are bonded or not. Related to this comment is that IQA acts on a supermolecular electron density. Put differently, there is no trace of any ideas of long‐range (intermolecular) perturbation theory within the IQA framework. This fact is an advantage in the design of force fields, as is the case for the QCT‐based force field^39^ called FFLUX.^32^

Equation (1) demonstrates that the total energy of a system is fully described as a sum of single‐atom and pairwise energy contributions,

(1) $$E_{\mathrm{Total}-\mathrm{IQA}}=\sum_{A}^{n}E_{\mathrm{intra}}^{A}+\sum_{A}^{n}\sum_{B<A}^{n-1}E_{\mathrm{inter}}^{AB}$$

where *n* is the number of atoms in the system. The physical quantities $E_{\mathrm{intra}}^{A}$ and $E_{\mathrm{inter}}^{AB}$ can be further decomposed into electrostatic, exchange and correlation terms. Note that the latter two types of energy terms, whilst physical distinguishable, are lumped together when using^40^ DFT. Because this work focuses on intra‐atomic energies, we will not look further into the decomposition of inter‐atomic energies. The intra‐atomic energies can be broken down into Coulombic and kinetic energy contributions, as shown in Equation (2):

(2) $$E_{\mathrm{intra}}^{A}=V_{\mathrm{ne}}^{AA}+V_{\mathrm{ee}}^{AA}+T^{A}$$

where $V_{\mathrm{ne}}^{AA}$ describes the interactions between the nucleus and electrons of a single atom *A*, $V_{\mathrm{ee}}^{AA}$ describes the interactions between electrons in that atom, and $T^{A}$ describes the kinetic energy of the electrons belonging to *A*. The absolute values of these energy contributions represent the energy released in building an atom from isolated electrons and the nucleus, all starting from infinite separation. These energies are enormous, that is, of the order of hundreds of thousands of kJ/mol for a second‐row element, and thus not within a chemical scale. It is therefore much more useful to measure changes in these contributions as the atom moves from one system to another, invoking the inherent energetic transferability of topological atoms. As a quick aside we note that T^A^ is the kinetic energy associated with the Kohn‐Sham fictitious system, directly built from Kohn‐Sham orbitals. This quantity does not include the so‐called correlation kinetic energy. However, as we will see in the figures of the Results and Discussion section, there are large variations in the values of T^A^ upon the geometry changes induced in this study. Hence, we assume that the correlation kinetic contribution will have only a negligible impact on these graphs.

In this paper, we investigate systems wherein two molecules are brought closer and closer together, resulting in compression in the volumes of the frontier atoms. This deformation of the frontier atoms is associated with an energy penalty described by the atomic deformation energy $E_{\mathrm{def}}^{A}$ , which is calculated by subtracting the intra‐atomic energy of the atom in the free molecule from the intra‐atomic energy of that atom in the system [Eq. (3)]:

(3) $$E_{\mathrm{def}}^{A}=E_{\mathrm{intra}(\mathrm{in}\;\mathrm{system})}^{A}-E_{\mathrm{intra}(\mathrm{free})}^{A}$$

The deformation of intra‐atomic energy can be decomposed into deformations of $V_{\mathrm{ne}}^{AA}$ , $V_{\mathrm{ee}}^{AA}$ and $T^{A}$ by subtracting the “free” value from the in‐system value in the same fashion, for example as shown in Equation (4):

(4) $$T_{\mathrm{def}}^{A}=T_{\mathrm{in}\mathrm{system}}^{A}-T_{\mathrm{free}}^{A}$$

The total repulsion between the two atoms is then represented as a sum of the deformations of their intra‐atomic energies [Eq. (5)]:

(5) $$E_{\mathrm{def}}^{AB}=E_{\mathrm{def}}^{A}+E_{\mathrm{def}}^{B}$$

Note that hereafter the subscript is dropped as all energies discussed are understood to be deformations. Thus one should be clear about interpreting a “negative kinetic energy” as always referring to a *change* in kinetic energy (i. e. a deformation). This description of short‐range repulsion as a deformation of intra‐atomic energies, rather than an inter‐atomic energy, is subtle but is important for characterising the interaction as steric. This interpretation is also not without precedent, as short‐range steric repulsion and deformation are often used interchangeably.^11^,^41^,^42^ Note that, while we adhere to the interpretation of steric energy laid out in Equation 5, we also regularly look at the two terms of Equation 5 separately in order to characterise the behaviour of individual atoms. This action is necessary when looking at asymmetric systems in which the summation of deformation energies will result in the loss of information about the behaviour of the individual atoms. This being said, all fits performed are for the summed deformation energy as this is the physically meaningful short‐range repulsion.

### 2.2 Repulsive Potential

Paper I established that short‐range exchange repulsion in IQA is better represented by a Buckingham‐type potential^2^ as opposed to the widely used Lennard‐Jones potential. As such our work will focus only on the Buckingham potential [Eq. (6)]

(6) $$V^{Buckingham}=A\exp\left(-Br\right)$$

where *A* and *B* are fitted constants (not be confused with the use of the same letters *A* and *B* for topological atoms) and *r* is an internuclear distance. Equation 6 shows only the repulsive part of the potential because this is the only term relevant to this work. This equation was used for all fits.

### 2.3 Computational Details

GAUSSIAN09^43^ was used to calculate optimised geometries for single molecules and generate wavefunctions for all molecules and systems at the B3LYP/aug‐cc‐pVTZ level of theory. IQA calculations were performed with version 17.11.14 of the program AIMALL^44^ to extract the intra‐atomic energies and the contributing energies (*T*, *V_ne_*, and *V_ee_*). Charge and volume information were also calculated by AIMALL by respectively integrating the electron density and the uniform unity function over the volume of the topological atom. In the first and largest batch of experiments, the geometry‐optimised “free” monomers were used as reference molecules while the dimer systems, at various intermolecular separations, were constructed using these monomers’ geometries. Note that a smaller series of experiments focused on the effect of geometry relaxation, and thus did not freeze the monomeric geometries. Wavefunctions were then calculated for each system at each separation and analysed using AIMALL. The dimer systems were not re‐geometry‐optimised so as to better control the orientation of approach of the monomers.

**Figure 1:**
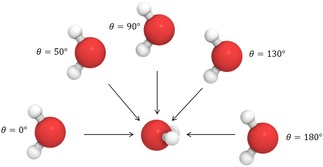
A sample of orientations of geometry‐optimised water dimers in an exemplar theta scan. For each orientation, one water molecule approaches the central water along the coordinate indicated by an arrow. The molecular images were produced using QuteMol.^45^

Paper I investigated uncluttered approaches of atoms, wherein molecules were brought together linearly to prevent unwanted interference of substituents with the atoms of interest. Using this methodology, an exponential relationship between separation and deformation energy was found in all cases. We sought to expand this model by including more diverse orientations of approach in N_2_, O_2_, F_2_, NH_3_, H_2_O, and HF dimers. To achieve this, we represented the two monomers in a polar coordinate system, thereby introducing the polar angle θ (theta). Figure 1 illustrates the way orientations are controlled for the water dimer. We defined an initial orientation as a staggered conformation with respect to the hydrogen atoms where the two heavy atoms faced each other. The relative orientation of the molecules was altered by keeping one molecule fixed and moving the other by varying θ from $0^{\circ }$ to $180^{\circ }$ in increments of $10^{\circ }$ . We will hereafter call this examination a “theta scan”. For each of the θ‐controlled orientations, we then brought the monomers together by systematically decreasing the internuclear separation of the main‐group atoms denoted *X* (where X=N, O, or F).

**Figure 2:**
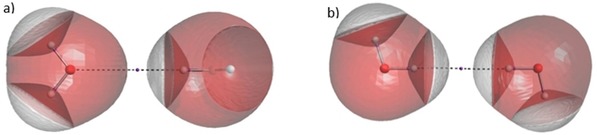
IRIS visualisation^46^,^47^ of the H_2_O dimer in the linear H−O approach (a); IRIS visualisation of the H_2_O dimer in the linear H−H approach (b).

The results of the theta scan experiments raised questions about the behaviour of hydrogen atoms in different environments, so we designed new experiments to categorise and explain our observations. In these experiments, a hydrogen atom in one molecule was made to approach an atom in another molecule in a linear fashion. These experiments are split into two categories: hydrogen‐X approaches and hydrogen‐hydrogen approaches, which are demonstrated in Figures 2a and 2b, respectively. Substituents were positioned so as to maximise the distance between them and the approaching atoms. Such configurations minimise their influence.

We varied the environment of the hydrogen atom by changing the atoms to which hydrogen was bonded and which it was approaching. For example, a hydrogen atom bears a positive charge^48^ when bonded to a nitrogen atom but is largely neutral when bonded to a carbon atom. As discussed later, the charge of the hydrogen atom has a significant effect on the behaviour of its intra‐atomic energy. We focus predominantly on second‐row elements in this paper but a full list of systems studied, which includes third‐row elements also, can be found in the Supporting Information.

As an extension to these experiments, we performed a selection of them again but this time allowing the geometries of the molecules to partially re‐geometry‐optimise during the approach. We fixed the minimum number of coordinates to preserve the approach orientation but allowed the internal coordinates of each molecule to vary. In practice this meant fixing the bond length between approaching atoms at each separation as well as fixing some dihedral angles. Using this method we did a theta scan for HF in which bond lengths were allowed to change, as well as hydrogen‐X and hydrogen‐hydrogen experiments with H_2_O and NH_3_ in which both bond lengths and internal bond angles were allowed to vary.

IQA‐calculated atomic volumes are bounded, at the interior by an interatomic surface, and at the exterior by an iso‐electron‐density envelope at a certain value, typically set at 0.001 a.u. The atomic volumes are therefore a measure of the size of an atom's electron cloud and are element‐dependent. The repulsion experienced between two atoms should depend on the distance between their electron clouds rather than on internuclear distance, so it is practical to compare systems with atoms at separations relative to the size of their electron clouds. The reasoning behind this is detailed in Paper I. For convenience we used Bondi's elemental van der Waals radii^49^ as surrogates for the size of atomic electron clouds. We begin with an internuclear separation for the atoms of interest equal to 130 % of the sum of their van der Waals radii. We then decreased the separation to 70 % in increments of 4 %.

The upper separation limit was chosen as the trends in the data disappeared beyond it. In other words, a data extrapolation beyond 130 % of the sum of the van der Waals radii showed that all IQA energy contributions tend towards their values in the reference molecule. The lower limit was chosen because at close separations, substituent hydrogen atoms begin to interact more with the atoms of interest and the interaction energy of the atoms of interest are no longer isolable. This is especially true for more sterically crowded systems such as the NH_3_ dimer.

## 3 Results and Discussion

### 3.1 Theta Scan

For each theta increment we fitted a Buckingham potential to the sum of the deformation energies of the two heavy atoms. Table 1 shows the details of the fits for the HF dimer, while information for the other dimers studied is provided in the Supporting Information in Tables S1–S6.

**Table 1:** All of the relevant information for the HF dimer theta scan. Absolute root‐mean‐square (RMS) errors in kJ/mol for the fits from $\theta =0^{\circ }\;to\;180^{\circ }$ for the sum of the deformation energies of the main group atoms. The minimum, maximum and range of energies in kJ/mol are provided to give context to the absolute RMS errors. The unit of coefficient *A* is kJ/mol and that of *B* is in Å^−1^.

Theta	RMS error	Energy range	Minimum energy	Maximum energy	Coefficient A	Coefficient B
0	1.9	147.4	4.5	151.9	22 194	2.4299
10	1.9	148.9	4.4	153.3	22 841	2.4393
20	1.9	153.2	4.3	157.5	24 520	2.4604
30	1.8	160.1	4.1	164.2	26 693	2.4810
40	1.6	168.7	3.8	172.5	29 010	2.4967
50	1.3	177.8	3.4	181.3	31 321	2.5088
60	1.0	186.3	3.0	189.3	33 936	2.5258
70	0.8	193.4	2.4	195.8	37 222	2.5533
80	0.6	199.1	1.8	201.0	41 176	2.5888
90	0.5	205.2	1.4	206.6	45 373	2.6218
100	0.5	214.4	1.3	215.7	48 432	2.6321
110	0.5	230.2	1.9	232.0	48 766	2.6005
120	0.9	255.4	3.3	258.7	45 998	2.5204
130	1.5	293.5	5.6	299.2	42 918	2.4164
140	1.7	347.5	8.1	355.6	42 680	2.3291
150	2.1	427.1	9.1	436.1	63 271	2.4174
160	3.6	405.5	7.8	413.3	99 405	2.6662
170	2.4	9.1	5.7	14.8		
180	5.0	41.8	−37.7	4.0

**Figure 3:**
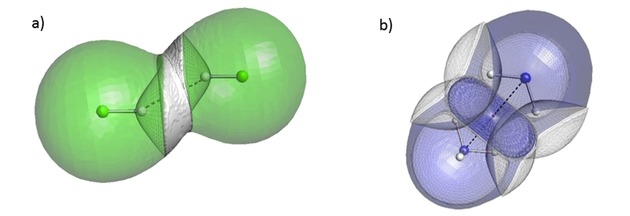
IRIS visualisation of the HF dimer at $\theta =160^{\circ }$ (a). This demonstrates the lack of compression of the atomic volumes of the fluorine atoms. IRIS visualisation of the NH_3_ dimer at $\theta =180^{\circ }$ (b). This demonstrates the compression of the atomic volumes of the nitrogen atoms.

The absolute RMS errors for the fits in Table 1 are all low but, when considering them in the context of the energy ranges, it is evident that the quality of fits deteriorates at the largest values of θ . For example, at $\theta =170^{\circ }$ , the RMS error is 26.4 % of the energy range. It is therefore pertinent to ask here if the interaction between two heavy atoms, when blocked by hydrogens, can still be considered as a steric one. We define a steric interaction as a mutual compression of atomic volumes that occurs through‐space. Figure 3a shows that at $\theta =160^{\circ }$ for the HF dimer, there is no through‐space compression of the fluorine atomic volumes. This accounts for the worse fits for HF at the largest theta values as there is clearly no steric interaction between the fluorine atoms. In contrast, the fits for all other dimers examined remain of good quality throughout the theta scan, as evidenced in the Supporting Information. Inspection of the water and ammonia dimers at $\theta =180^{\circ }$ shows that the heavy atoms still touch each other. In other words, there is still a significant portion of non‐bonded electron density between the hydrogen atoms that can interact through‐space. An example of this situation is shown in Figure 3b. As a result, we have no problem characterising this as a steric interaction, although it is likely perturbed by “contaminating” interactions with the hydrogen atoms. Overall, these fits demonstrate that IQA recovers a Buckingham potential without any problems, not just for the most basic (of Paper I) of approaches but for a wide range of approaches.

**Figure 4:**
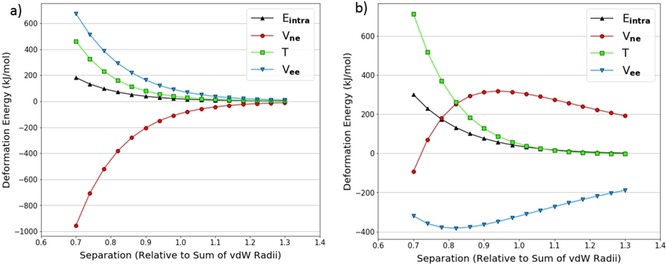
Decomposed intra‐atomic energy for the nitrogen atoms at $\theta =90^{\circ }$ in the N_2_ dimer theta scan (a); decomposed intra‐atomic energy for the nitrogen atoms at $\theta =10^{\circ }$ in the NH_3_ dimer theta scan (b).

We also looked at the decomposition of the intra‐atomic energy into its constituent terms: V_ne_, V_ee_, and T. An example characteristic of a pure volume compression (in the N_2_ dimer) is shown in Figure 4a. The behaviour of the energy contributions is as expected. The kinetic energy *T* becomes more positive upon compression because the electrons are progressively confined, which increases their momentum, as required by the uncertainty principle. Again upon compression, the inter‐electron potential energy V_ee_ becomes increasingly positive as electrons end up closer together on average and thus experience greater repulsion. The (attractive and negative) nuclear‐electron potential energy V_ne_ becomes increasingly negative as electrons are on average closer to the nucleus and so experience greater attraction. We also see that V_ne_ and V_ee_ are approximately equal and opposite, so the intra‐atomic energy is dominated by the kinetic energy term.

On the other hand, Figure 4b shows a similar plot for the nitrogen atoms in the NH_3_ dimer at $\theta =10^{\circ }$ . Here the V_ee_ and V_ne_ terms can no longer be explained by only considering a volume compression. The nitrogen atoms become more positive as they approach each other; this effect contrasts to that in the case of the neutral nitrogen atoms in the N_2_ dimer (Figure 4a). This intramolecular depletion of charge on the nitrogen atoms (in NH_3_) means that V_ne_ will become positive (except for the shortest separation) because the electron‐nuclear attraction is reduced while V_ee_ will become negative because electron‐electron repulsion is reduced. Note that all energies discussed are actually deformation energies, that is, differences between a state and its reference. As such it is evident that, at the largest separations, the trends in V_ne_ and V_ee_ are consistent with a charge transfer. As the separation decreases below 0.9 times the sum of the van der Waals radii, the trends switch direction suggesting that volume compression effects take over the behaviour.

In spite of the nuances in the behaviour of V_ne_ and V_ee_, the intra‐atomic energy is exponential in both Figures 4a and 4b. This is because V_ne_ and V_ee_ mostly cancel each other out leaving the kinetic energy to dominate the intra‐atomic energy. Thus we conclude that charge transfer effects are not of great importance for heavy atoms.

Inspection of the plots of the deformation energy of hydrogen and its contributing terms discovered behaviour that deviated significantly from the volume‐compression model, wherein the deformation energy was often negative. As a result this behaviour was more rigorously investigated in the linear dimer experiments.

### 3.2 Hydrogen‐X Approaches

For all hydrogen‐X approaches tested, the sum of the deformation energies of the approaching atoms was well represented by an exponential function. The RMS errors displayed in Table 2 are all within an acceptable range given the scale of the energies. Further details on these fits, for all of the systems studied, can be found in Table S7 in the Supporting Information.

**Table 2:** Root‐mean‐square errors in kJ/mol for the exponential fits for some of the hydrogen‐X approaches studied.

System	RMS error	System	RMS Error	System	RMS error
NH_3_−NH_3_	2.7	FH−FH	1.1	OH_2_−OH_2_	2.5
NH_3_−OH_2_	2.5	FH−OH_2_	6.1	OH_2_−NH_3_	2.7
NH_3_−FH	1.8	FH−NH_3_	1.9	OH_2_−FH	1.8

The series of approaches in Table 3 provide some insight into the potential physical meaning of the fit coefficients of Equation 6: the pre‐exponential factor *A* and the exponential factor *B*. Mathematically speaking, *A* sets the scale of the plot while *B* sets the gradient. Therefore it would make sense for *B* to correspond to the ‘hardness’ of an atom because the gradient gives a measure of the energy penalty incurred as the electron cloud is deformed. Indeed, the harder an atom is, the greater the expected energy penalty, for a constant change in r, in the comparison of two atoms. The trend in *B*, shown in Table 3, supports this idea because *B*, and so the gradient of the intra‐atomic energy, increases from nitrogen to fluorine. This would suggest that nitrogen is the ‘softest’ of the three atoms and fluorine the ‘hardest’, which is in agreement with chemical intuition and the literature.^50^, ^51^, ^52^ This trend is seen more than once in our work; further details are shown in the Supporting Information in Table S7. This finding is somewhat preliminary at this point but this exciting speculation could form the basis of future work dedicated to hardness.

**Table 3:** Complete fit details for a series of H−X experiments in which a CH_4_ hydrogen atom approaches nitrogen, oxygen and fluorine atoms.

System	RMS error	Energy range	Minimum energy	Maximum energy	Coefficient A	Coefficient B
CH_4_−NH_3_	1.4	133.9	9.4	143.3	4 579	1.8071
CH_4_−OH_2_	1.3	111.2	7.1	118.3	4 457	1.9144
CH_4_−FH	1.1	81.6	4.5	86.1	4 460	2.1216

**Figure 5:**
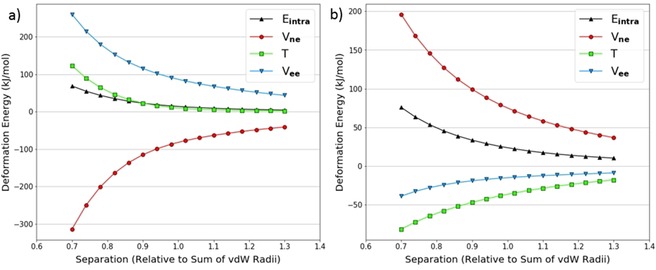
Decomposed intra‐atomic energy for the nitrogen in a NH_3_ dimer hydrogen‐nitrogen approach (a); decomposed intra‐atomic energy for the hydrogen in a NH_3_ dimer hydrogen‐nitrogen approach (b).

The fits in Tables 2 and 3 are all of good quality. However, when the intra‐atomic energies of each individual atom are decomposed into V_ne_, V_ee_, and T, it is clear that the dominant effects governing the behaviour of the heavy atom (Figure 5a) and the hydrogen atom (Figure 5b) energy contributions are different. The energy contributions displayed for nitrogen in Figure 5a are consistent with a volume compression as shown in Figure 4a, and the intra‐atomic energy is dominated by kinetic energy. While the intra‐atomic energy of hydrogen in Figure 5b is still positive and exponential, the kinetic energy is negative and the dominant term is now V_ne_.

**Figure 6:**
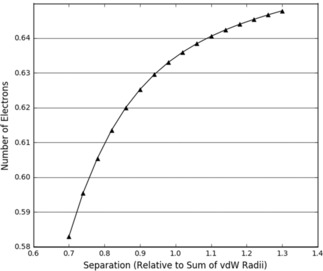
The change in the number of electrons for the hydrogen in the NH_3_ dimer hydrogen‐nitrogen approach.

The hydrogen atom becomes increasingly positive during the approach, as shown in Figure 6. This means that the average number of electrons in the atomic volume decreases. We therefore expect that T will become more negative as there are fewer electrons to add to the total kinetic energy. V_ne_ and V_ee_ are also expected to behave as explained above in reference to Figure 4b. These expectations are met but, in this case (as opposed to the case in Figure 4b) the trends are consistent throughout the scan suggesting that this is characteristic entirely of a charge transfer despite the compression of atomic volume.

### 3.3 Hydrogen‐Hydrogen Approaches

**Figure 7:**
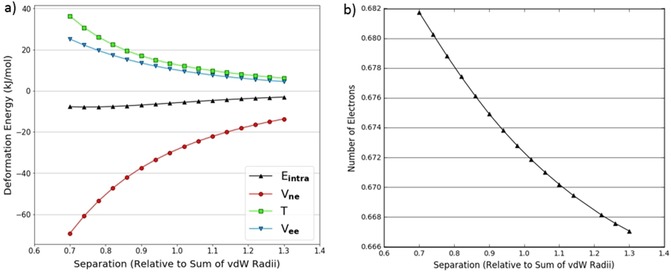
Decomposed intra‐atomic energy for a hydrogen atom in a NH_3_ dimer hydrogen‐hydrogen approach (a); change in number of electrons within the volume of a hydrogen atom in a NH_3_ dimer's hydrogen‐hydrogen approach (b).

To further investigate the behaviour of hydrogen we looked at a hydrogen approaching hydrogen in various dimers. An example of the energy contributions for one of the interacting hydrogen atoms in the NH_3_ dimer is shown in Figure 7a.

Figure 7a demonstrates trends in the energy contributions that are characteristic of a volume compression. Figure 7b shows the atom also gains 0.015 electrons over full compression (from maximum to minimum separation), which reinforces the effects of compression on the energy contributions. This is the reverse of what was seen in Figure 5b, where the volume and charge effects oppose each other. In contrast to other atoms in which behaviour is dominated by volume compression, the V_ne_ contribution is of sufficient magnitude to cancel out the T and V_ee_ contributions resulting in a near‐zero intra‐atomic energy.

**Figure 8:**
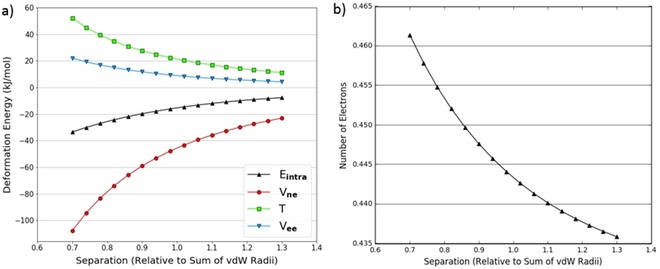
(Decomposed intra‐atomic energy for a hydrogen atom in a H_2_O dimer hydrogen‐hydrogen approach (a); change in number of electrons for a hydrogen atom in a H_2_O dimer hydrogen‐hydrogen approach (b).

Figures 8a and 8b show the same approach for H_2_O. Here there is a gain of about 0.027 electrons in going from maximum to minimum separation, which is almost twice as much as in the NH_3_ system. This means that the V_ne_ contribution is larger relative to T and V_ee_, and therefore the intra‐atomic energy is decidedly negative. Although this negative intra‐atomic energy does not match the repulsive nature of a steric interaction, the literature often assumes that hydroxyl and amine^53^ hydrogen atoms provide no steric hindrance. For example, in many water models^54^, ^55^, ^56^, ^57^, ^58^, ^59^ a zero collision diameter is assigned to the Lennard‐Jones potential for hydrogen atoms.

**Figure 9:**
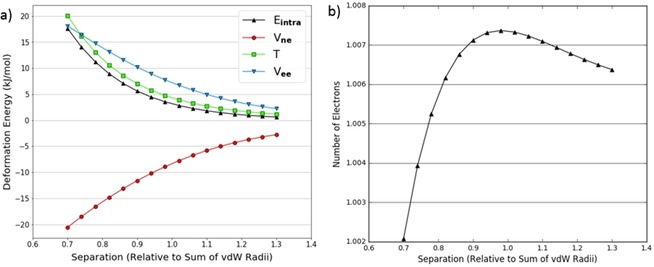
Decomposed intra‐atomic energy for a hydrogen atom in a CH_4_ dimer hydrogen‐hydrogen approach (a); change in number of electrons for a hydrogen atom in a CH_4_ dimer hydrogen‐hydrogen approach (b).

In CH_4_, the hydrogen atom is essentially neutral and undergoes a very small change in charge (less than 0.006 electrons) during the experiment, as shown in Figure 9b. The magnitude of V_ne_ is therefore not affected very much, and the trends in the energy contributions shown in Figure 9a are consistent with a typical volume compression. Specifically, it is now T that dominates the intra‐atomic energy because V_ne_ and V_ee_ mostly cancel each other out.

No concern should arise by the fact that B3LYP was used to model the methane dimer. Indeed, this complex is mainly held together by dispersion, which B3LYP cannot describe. However, as it is the short‐range contribution that is at work in steric hindrance, B3LYP is still reliable enough.

We have now observed a spectrum of different hydrogen behaviours, which demonstrate how each of the energy contributions responds differently to changes in volume and charge. The kinetic energy is influenced significantly by volume but is relatively resistant to changes in atomic charge. The increasing confinement of electrons during a compression inevitably increases their kinetic energy as a result of the uncertainty principle. This increase will not be greatly affected by the loss of electrons provided there is a sufficient base population of electrons being compressed. However, V_ne_ and V_ee_ are naturally predominantly dependent on electronic charge as they both have significant Coulombic contributions. However, V_ne_ and V_ee_ will be affected somewhat by volume compression as the average electron‐nuclear and electron‐electron distances decrease. Generally there is an inverse symmetry in the behaviour of V_ne_ and V_ee_, which results in a large degree of cancellation when summed. However, they do not perfectly cancel each other out because V_ee_ is typically more vulnerable to charge transfer than V_ne_. We believe that this is because V_ee_ is purely electron dependent whereas V_ne_ also depends on the nuclear charge, which is a fixed quantity. The lack of cancellation can lead to V_ne_ dominating the intra‐atomic energy at lower electron populations when the kinetic energy is not of sufficient magnitude to compensate for the difference between V_ne_ and V_ee_.

The evidence for this model is best demonstrated by further examining the H_2_O and CH_4_ dimers, which exhibit the extremes of hydrogen behaviour. In Figure 8a (H_2_O dimer) the hydrogen intra‐atomic energy is clearly dominated by V_ne_ whereas in Figure 9a (CH_4_ dimer), the interplay between energy terms is markedly different. We explain this difference in behaviour by considering that hydrogen's electron density is provided entirely by valence electrons. This makes hydrogen far more vulnerable than heavier atoms to changes in atomic charge. In CH_4_, the hydrogen begins with a charge of −0.006 e which means it has 1.006 electrons. We can see from Figure 9b that the number of electrons decreases by about 0.005. Because this is only a minor effect and because hydrogen still has sufficient electron density, the compression of volume results in a positive deformation of the kinetic energy. This is contrasted with the hydrogen atom in H_2_O, which is positive and begins with a charge of +0.56 e meaning it has 0.44 electrons. Figure 8b shows that this hydrogen gains about 0.03 electrons towards full compression. This is a much greater change than in the CH_4_ case which, coupled with the fact that the hydrogen begins with a smaller electron density, means that the magnitudes of the V_ee_ and T are significantly reduced relative to V_ne_.

The model presented here applies not only to hydrogen but also to heavier atoms. In contrast to hydrogen, heavy atoms have a larger population of electrons because they have a core of electrons. This means that they are not so affected by the gain or loss of valence electrons because, despite these fluctuations, the core electron density will behave consistently under compression. As such, the kinetic energy is always sufficiently positive such that it dominates the intra‐atomic energy despite the incomplete cancellation of V_ne_ and V_ee_. This means that the intra‐atomic energy is always well represented by a Buckingham potential in a steric interaction.

**Figure 10:**
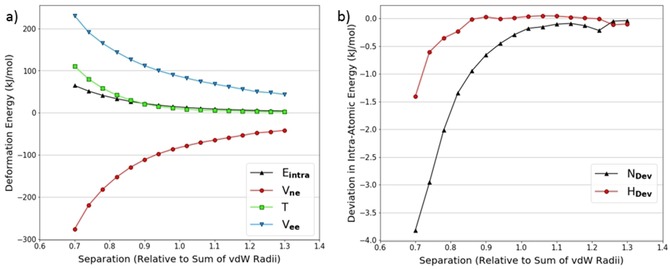
(left, a) Decomposed intra‐atomic energy for the nitrogen atom in a relaxed NH_3_ dimer hydrogen‐nitrogen approach; (right, b) Intra‐atomic energy deviations from the equivalent rigid experiment shown in Figure 5a. The deviation is calculated as relaxed minus rigid.

In addition to the experiments presented so far, we performed a few relaxation experiments in which partial re‐optimisation of the geometries was allowed during the approaches. Comparison of Figures 10a and 5a shows that for the same experiment, allowing partial re‐optimisation does not have any effect on the trends in the energy contributions. Furthermore, Figure 10b demonstrates that the absolute deviations in the intra‐atomic energy for both the nitrogen and hydrogen atom in the scan are relatively small, only becoming significant at the smallest separations. This suggests that our model still holds when molecules are allowed to relax, which is promising because this scenario is closer to reality. Note that further evidence relating to these experiments can be found in Section 4 of the Supporting Information.

## 4 Conclusions

We have specified that for two atoms to be sterically interacting, they must experience a mutual, through‐space compression of atomic volume. This can occur in a wide range of approach orientations even when smaller substituents clutter the line of approach. We have shown that the deformation of the IQA intra‐atomic energy is responsible for stereo‐electronic effects. When the intra‐atomic energy is decomposed it is evident that these effects are produced by the interplay between the constituent energy contributions T, V_ne_ and V_ee_. T responds predominantly to volume deformation and so it appears to be the main contributor to the steric effects, whereas V_ne_ and V_ee_ respond far more strongly to charge transfer and so appear to comprise the main contribution to the electronic effects.

This interplay of energy contributions is similar for all heavy atoms across all situations, provided there is volume compression. However, the interplay of energy terms for hydrogen atoms is not constant across different situations. This is because hydrogen is more sensitive to changes in atomic charge as its electron density is provided entirely by valence electrons. As such, hydrogen's intra‐atomic energy is not necessarily well represented by a Buckingham potential and so hydrogen does not reliably produce steric hindrance, as is often assumed in the literature (e. g. parameterised water models). In summary, we provide for the first time evidence supporting this assumption.

## Conflict of interest

The authors declare no conflict of interest.

## Supporting information

As a service to our authors and readers, this journal provides supporting information supplied by the authors. Such materials are peer reviewed and may be re‐organized for online delivery, but are not copy‐edited or typeset. Technical support issues arising from supporting information (other than missing files) should be addressed to the authors.

Supplementary
