# Supplementary material for: Does the Intra‐Atomic Deformation Energy of Interacting Quantum Atoms Represent Steric Energy?
Source: ChemistryOpen. 2019 Feb 8;8(5):560–70. doi: 10.1002/open.201800275 (PMC6496634; doi:10.1002/open.201800275)
Supplement: Supplementary file 1 — Supplementary [file OPEN-8-560-s001.pdf]

Supporting Information

© Copyright Wiley-VCH Verlag GmbH & Co. KGaA, 69451 Weinheim, 2019

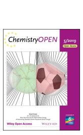

Does the Intra-Atomic Deformation Energy of Interacting Quantum Atoms Represent Steric Energy?

Benjamin C. B. Symons, Dominic J. Williamson, Campbell M. Brooks, Alex L. Wilson, and Paul L. A. Popelier*© 2019 The Authors. Published by Wiley-VCH Verlag GmbH & Co. KGaA. This is an open access article under the terms of the Creative Commons Attribution License, which permits use, distribution and reproduction in any medium, provided the original work is properly cited.An invited contribution to a Special Collection dedicated to Computational Chemistry

Supporting Information

1. Theta scans

1.1 NH_3 dimer

Table S1. Energy ranges and exponential fit data for NH_3 theta scan. Energy ranges and fit data are calculated from a sum of the data for both approaching atoms, whereas volume data pertains to a single atom.

Theta	RMS Error (kJ/mol)	Energy Range (kJ/mol)	Min Energy (kJ/mol)	Max Energy (kJ/mol)	Coefficient A (kJ/mol)	Coefficient B ($Å^{-1}$)	Change of Atomic Volume (%)
0	1.8	304.7	1.3	306.0	40,375	2.2475	-16.9
10	1.7	299.4	1.5	300.9	39,068	2.2402	-17.1
20	1.3	287.7	2.1	289.8	36,108	2.2218	-17.3
30	0.6	278.4	3.3	281.8	33,034	2.1951	-17.3
40	0.6	280.8	5.3	286.1	31,319	2.1654	-17.0
50	1.5	299.8	7.6	307.4	31,414	2.1356	-16.6
60	2.3	333.6	9.8	343.4	33,492	2.1148	-15.5
70	2.4	378.4	11.2	389.6	37,283	2.1056	-14.3
80	2.0	434.8	11.3	446.1	48,126	2.1595	-13.1
90	1.3	509.9	9.2	519.2	81,843	2.3319	-11.4

100	0.7	602.3	5.6	607.8	175,659	2.6104	-9.4
110	1.2	682.8	2.4	685.2	332,009	2.8483	-7.6
120	1.6	721.8	1.6	723.4	368,860	2.8716	-7.1
130	1.1	691.4	3.3	694.8	239,181	2.6907	-7.5
140	0.9	612.1	6.0	618.1	134,908	2.4829	-8.9
150	2.2	531.1	7.8	539.0	86,255	2.3420	-10.2
160	2.7	470.3	8.8	479.1	65,407	2.2702	-11.7
170	3.1	434.8	9.3	444.1	56,966	2.2422	-12.9
180	3.4	423.0	9.4	432.3	54,446	2.2345	-13.2

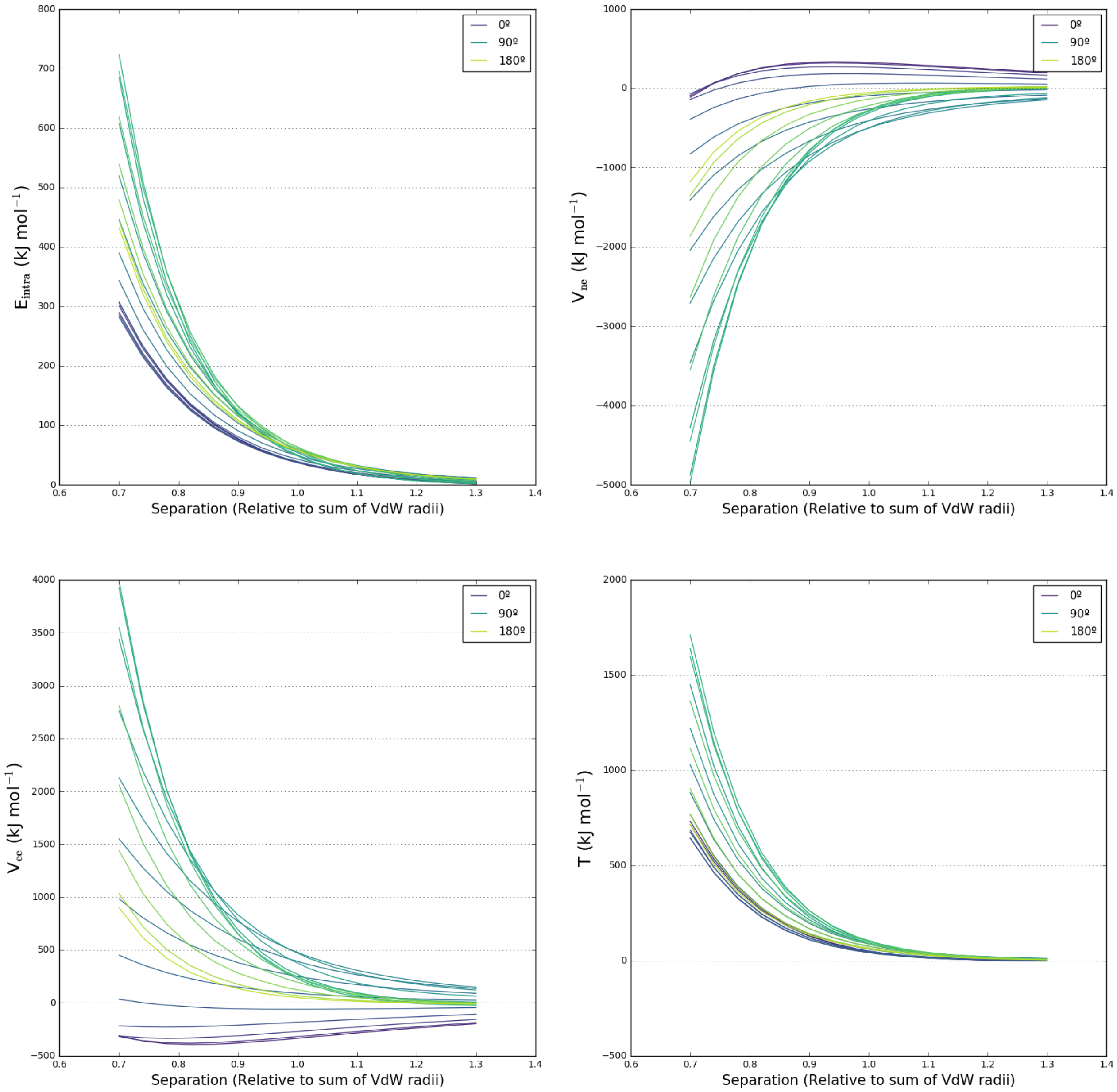

Figure S1. Intra-atomic energy and its contributions for all values of θ in the scan.

1.2 H_2O dimer

Table S2. Energy ranges and exponential fit data for H_2O theta scan. Energy ranges and fit data are calculated from a sum of the data for both approaching atoms, whereas volume data pertains to a single atom.

Theta	RMS Error (kJ/mol)	Energy Range (kJ/mol)	Min Energy (kJ/mol)	Max Energy (kJ/mol)	Coefficient A (kJ/mol)	Coefficient B (Å^{-1})	Change of Atomic Volume (%)
0	2.8	190.5	-3.2	187.3	65,662	2.7477	-9.9
10	2.6	191.3	-2.9	188.4	63,103	2.7267	-9.9
20	2.2	193.5	-2.1	191.4	56,842	2.6713	-10.1
30	1.6	197.3	-0.9	196.4	48,380	2.5850	-10.4
40	0.8	203.5	0.7	204.2	40,040	2.4799	-10.9
50	0.5	214.8	2.8	217.6	33,280	2.3657	-11.6
60	1.4	235.3	5.3	240.6	29,326	2.2612	-12.5
70	2.4	269.6	8.1	277.8	27,956	2.1733	-13.7
80	3.4	320.4	11.2	331.6	29,592	2.1177	-15.2
90	3.8	384.2	14.0	398.1	33,300	2.0860	-16.6
100	3.2	452.7	15.8	468.5	38,219	2.0703	-17.7
110	3.0	522.0	16.0	537.9	46,504	2.0925	-18.3
120	4.1	593.7	13.7	607.4	66,023	2.1973	-17.9
130	4.2	654.3	9.6	663.9	110,550	2.3980	-16.5
140	3.1	665.1	5.3	670.4	177,409	2.6174	-14.8
150	1.7	625.1	2.7	627.9	207,343	2.7235	-13.6
160	0.9	603.0	3.0	606.0	174,785	2.6627	-12.7
170	1.3	608.9	5.0	613.9	129,850	2.5178	-12.0
180	1.1	453.2	6.1	459.3	109,152	2.4336	-10.0

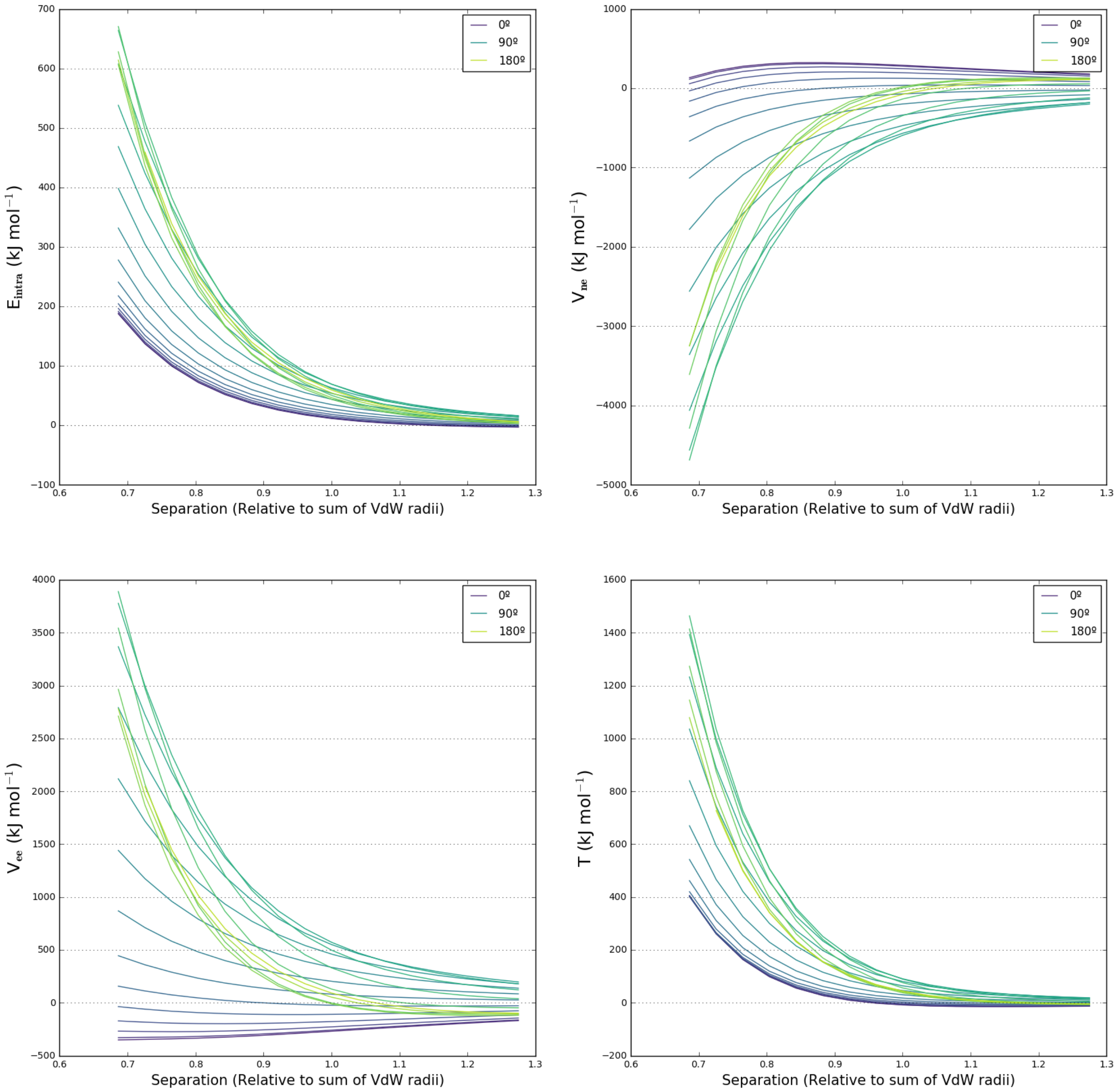

Figure S2. Intra-atomic energy and its contributions for all values of θ in the scan.

1.3 HF dimer

Table S3. Energy ranges and exponential fit data for HF theta scan. Energy ranges and fit data are calculated from a sum of the data for both approaching atoms, whereas volume data pertains to a single atom.

Theta	RMS Error (kJ/mol)	Energy Range (kJ/mol)	Minimum Energy (kJ/mol)	Maximum Energy (kJ/mol)	Coefficient A (kJ/mol)	Coefficient B ($Å^{-1}$)	Change of Atomic Volume (%)
0	1.9	147.4	4.5	151.9	22,194	2.4299	-7.6
10	1.9	148.9	4.4	153.3	22,841	2.4393	-7.7
20	1.9	153.2	4.3	157.5	24,520	2.4604	-7.8
30	1.8	160.1	4.1	164.2	26,693	2.4810	-8.0
40	1.6	168.7	3.8	172.5	29,010	2.4967	-8.1
50	1.3	177.8	3.4	181.3	31,321	2.5088	-8.5
60	1.0	186.3	3.0	189.3	33,936	2.5258	-8.7
70	0.8	193.4	2.4	195.8	37,222	2.5533	-9.0
80	0.6	199.1	1.8	201.0	41,176	2.5888	-9.3
90	0.5	205.2	1.4	206.6	45,373	2.6218	-9.7
100	0.5	214.4	1.3	215.7	48,432	2.6321	-10.2
110	0.5	230.2	1.9	232.0	48,766	2.6005	-10.6
120	0.9	255.4	3.3	258.7	45,998	2.5204	-11.0
130	1.5	293.5	5.6	299.2	42,918	2.4164	-11.5
140	1.7	347.5	8.1	355.6	42,680	2.3291	-11.5
150	2.1	427.1	9.1	436.1	63,271	2.4174	-11.5
160	3.6	405.5	7.8	413.3	99,405	2.6662	-12.1
170	2.4	9.1	5.7	14.8	74	0.5811	-11.8
180	5.0	41.8	-37.7	4.0	-14,673,600	5.3069	-11.1

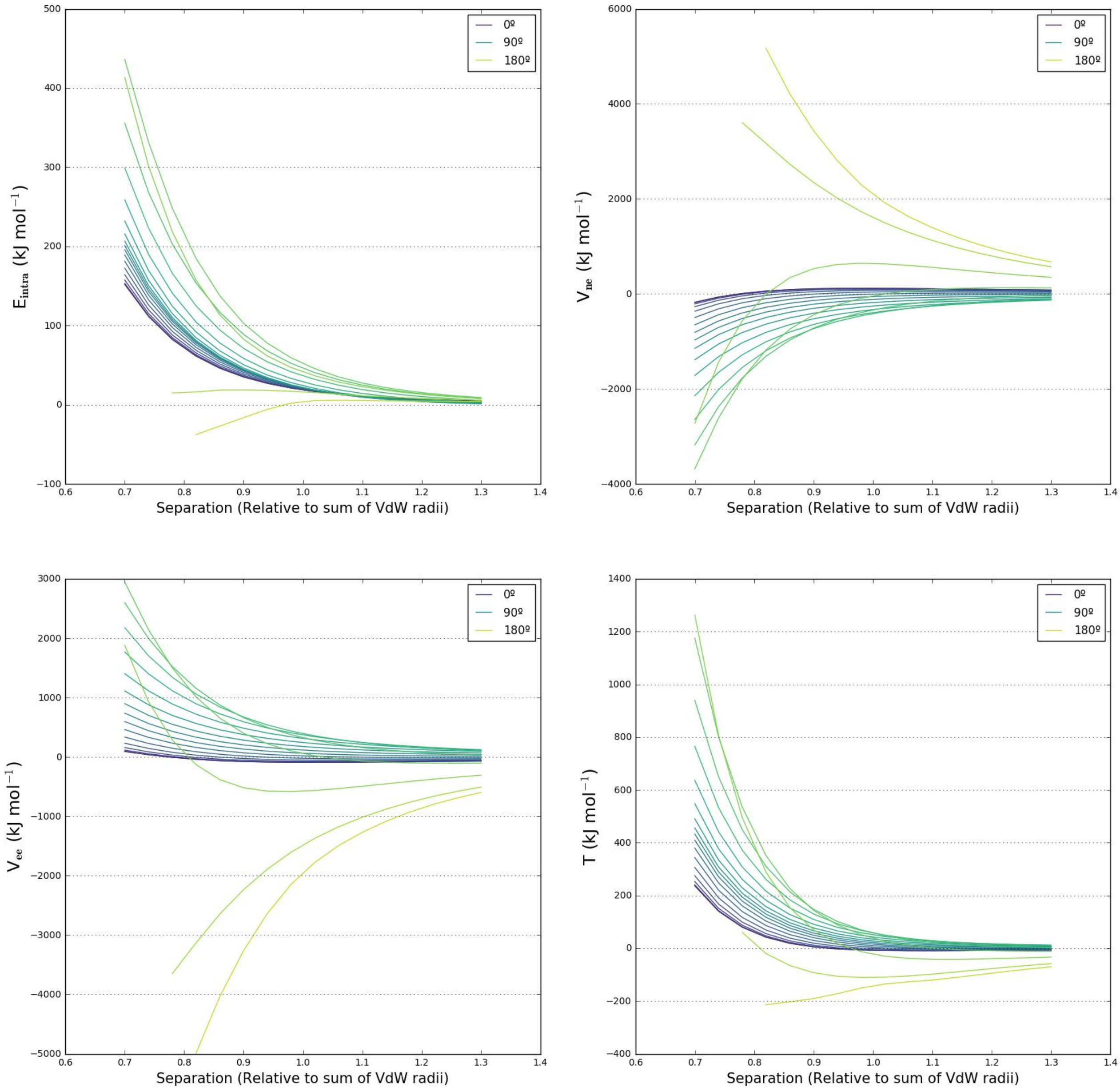

Figure S3. Intra-atomic energy and its contributions for all values of θ in the scan.

The deterioration of quality of the fits for the HF dimer theta scan discussed in the main text and shown in Tables 1 and S3 is reflected in some odd trends in the intra-atomic energy contributions at the largest values of θ shown in Figure S3. Note also that, while there is still significant volume compression of the fluorine atoms at the larger values of theta, we showed in the main text that this compression does not occur through-space and so the interaction is not steric.

1.4 N_2 dimer

Table S4. Energy ranges and exponential fit data for N_2 theta scan. Energy ranges and fit data are calculated from a sum of the data for both approaching atoms, whereas volume data pertains to a single atom.

Theta	RMS Error (kJ/mol)	Energy Range (kJ/mol)	Min Energy (kJ/mol)	Max Energy (kJ/mol)	Coefficient A (kJ/mol)	Coefficient B ($Å^{-1}$)	Change of Atomic Volume (%)
0	1.9	238.8	5.9	244.7	32,313	2.2556	-13.7
10	1.6	225.5	5.6	231.1	29,016	2.2313	-13.2
20	0.9	194.0	4.8	198.8	22,812	2.1878	-12.5
30	0.4	158.6	3.6	162.2	17,880	2.1673	-11.4
40	0.2	127.8	2.4	130.2	14,891	2.1831	-10.6
50	0.3	106.6	1.3	107.9	14,106	2.2437	-10.1
60	0.4	98.7	0.7	99.4	16,197	2.3454	-9.8
70	0.3	106.5	0.7	107.1	21,300	2.4378	-9.8
80	0.1	132.3	1.2	133.5	29,477	2.4876	-10.2
90	0.5	179.9	2.3	182.1	40,899	2.4969	-11.6

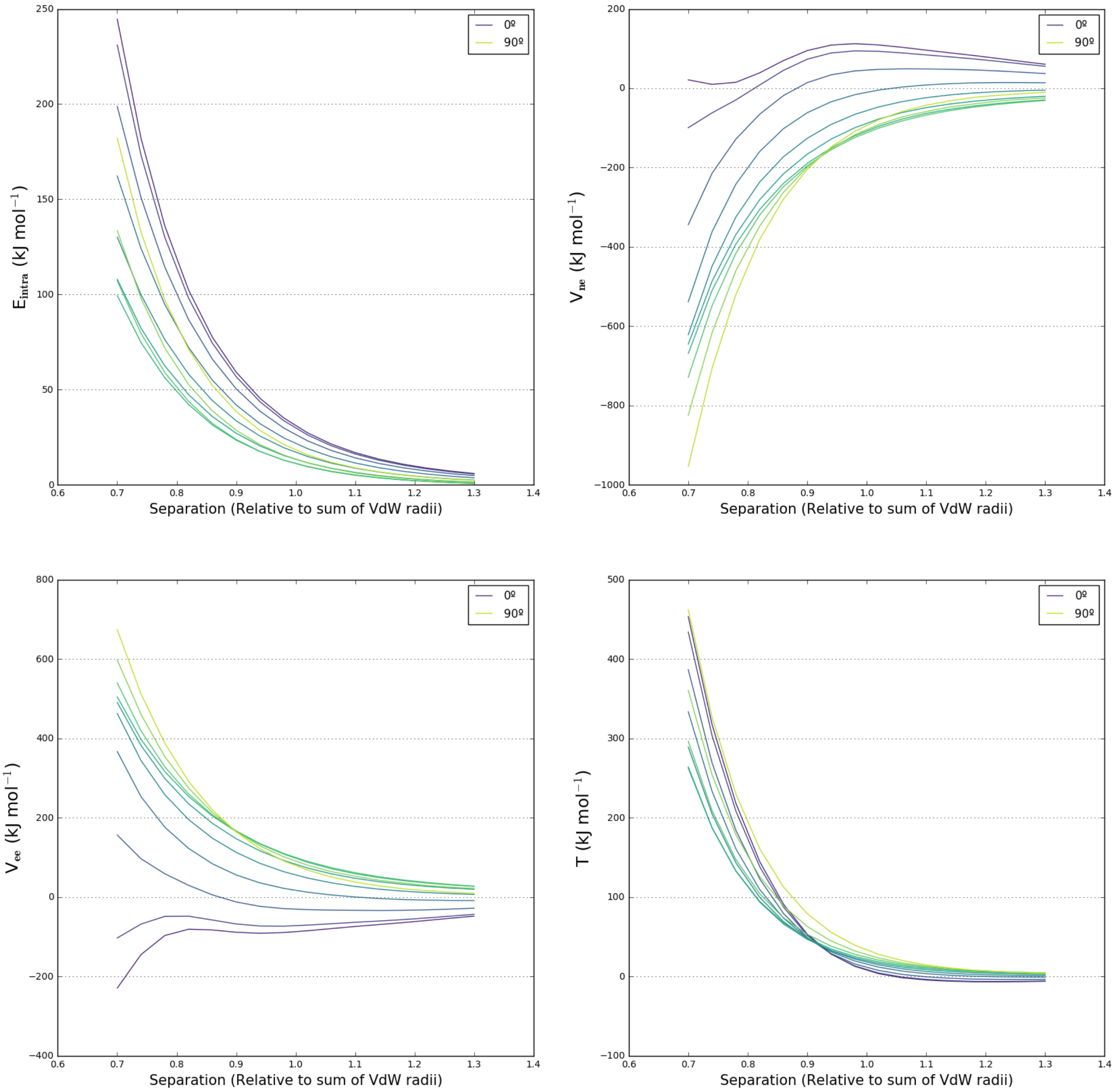

Figure S4. Intra-atomic energy and its contributions for all values of θ in the scan.

1.5 O_2 dimer

Table S5. Energy ranges and exponential fit data for O_2 theta scan. Energy ranges and fit data are calculated from a sum of the data for both approaching atoms, whereas volume data pertains to a single atom.

Theta	RMS Error (kJ/mol)	Energy Range (kJ/mol)	Min Energy (kJ/mol)	Max Energy (kJ/mol)	Coefficient A (kJ/mol)	Coefficient B (Å^{-1})	Change of Atomic Volume (%)
0	0.7	133.5	1.8	135.3	35,497	2.6205	-9.9
10	0.7	132.2	1.9	134.1	33,487	2.5973	-9.8
20	0.6	127.9	1.9	129.8	28,366	2.5346	-9.4
30	0.5	119.9	1.9	121.8	22,379	2.4524	-9.0
40	0.3	108.7	1.7	110.4	17,352	2.3781	-8.7
50	0.1	95.2	1.4	96.6	13,869	2.3345	-8.3
60	0.1	83.8	1.0	84.8	12,785	2.3575	-8.0
70	0.1	82.3	0.8	83.1	15,576	2.4598	-8.2
80	0.3	103.1	1.0	104.1	24,630	2.5709	-9.0
90	0.7	157.2	1.8	159.0	42,130	2.6253	-10.3

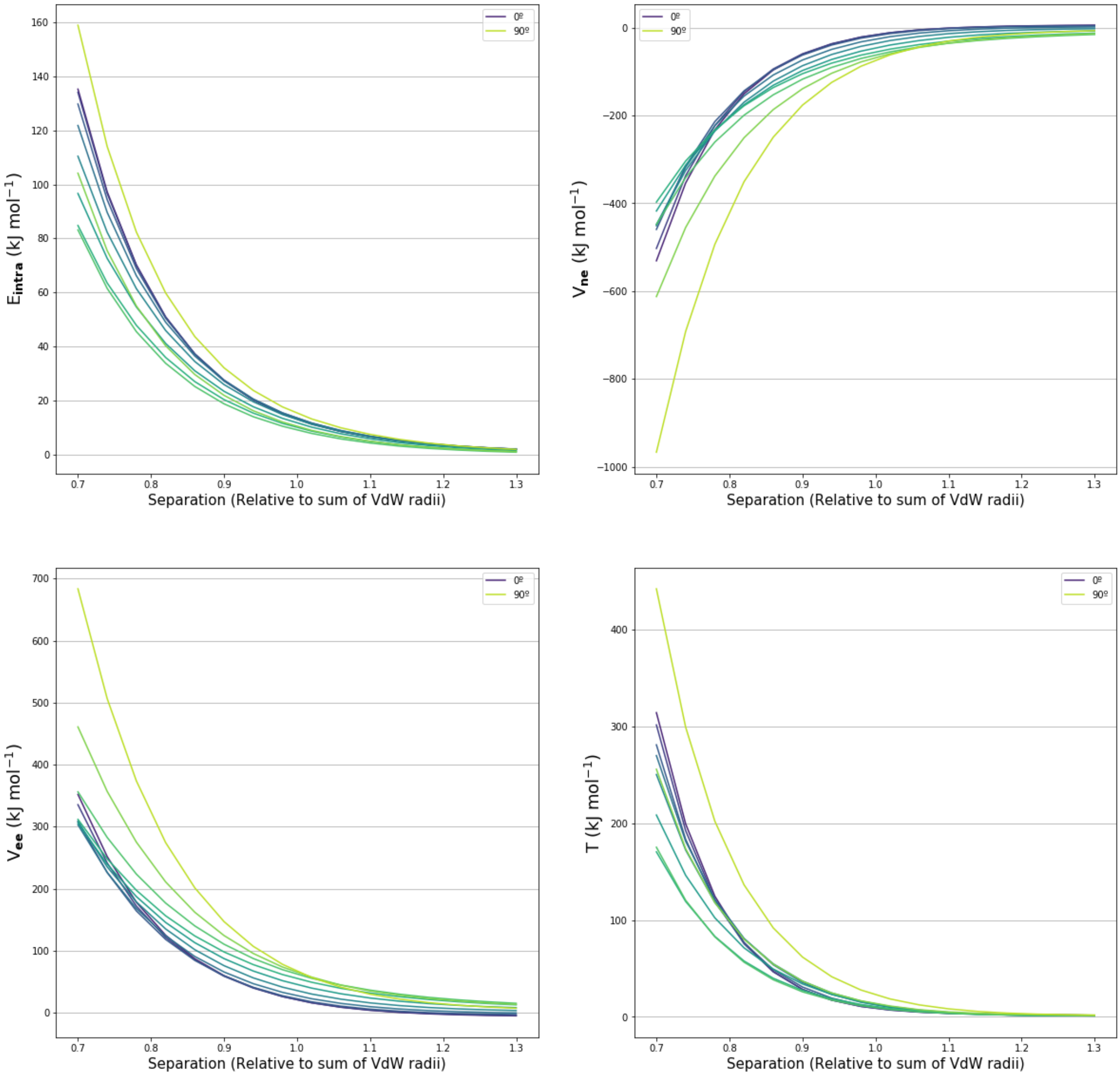

Figure S5. Intra-atomic energy and its contributions for all values of θ in the scan.

1.6 F_2 dimer

Table S6. Energy ranges and exponential fit data for F_2 theta scan. Energy ranges and fit data are calculated from a sum of the data for both approaching atoms, whereas volume data pertains to a single atom.

Theta	RMS Error (kJ/mol)	Energy Range (kJ/mol)	Min Energy (kJ/mol)	Max Energy (kJ/mol)	Coefficient A (kJ/mol)	Coefficient B (Å^{-1})	Change of Atomic volume (%)
0	0.8	79.6	-0.8	78.7	105,749	3.3160	-8.2
10	0.6	85.4	-0.5	84.9	83,975	3.1764	-8.1
20	0.2	94.5	0.2	94.6	47,655	2.8674	-8.1
30	0.2	96.5	0.9	97.5	24,974	2.5577	-7.9
40	0.3	90.1	1.6	91.6	14,055	2.3218	-7.4
50	0.3	78.0	1.8	79.8	8,667	2.1625	-6.8
60	0.2	66.0	1.7	67.8	6,321	2.0926	-6.5
70	0.2	63.0	1.5	64.5	6,835	2.1517	-7.0
80	0.3	81.5	1.3	82.8	13,320	2.3447	-8.3
90	0.8	143.7	1.4	145.1	41,188	2.6069	-10.3

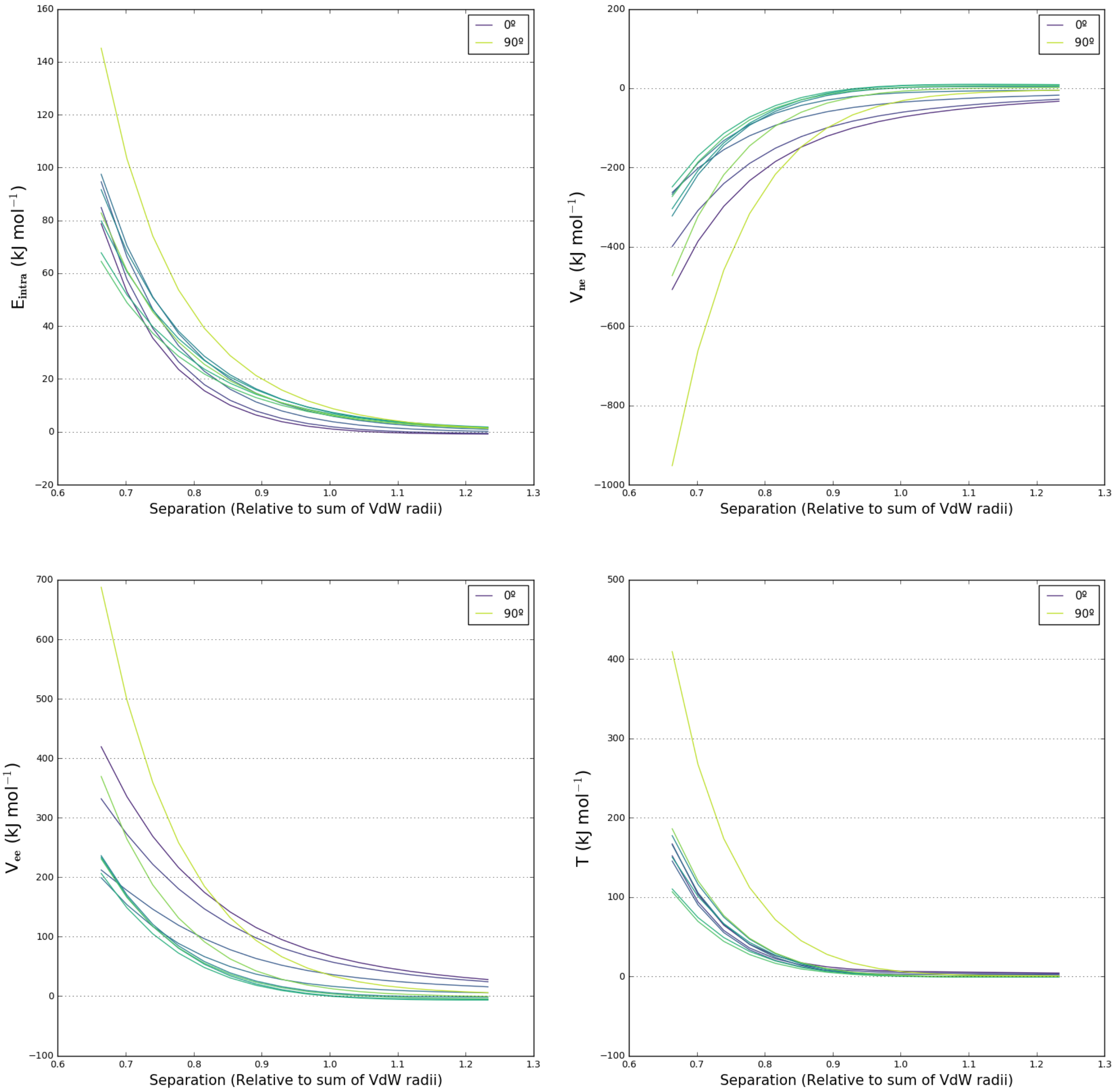

Figure S6. Intra-atomic energy and its contributions for all values of θ in the scan.

2. Hydrogen-bond type approaches (H…X)

Table S7. Energy ranges and exponential fit data for all H…X type approaches. Energy ranges and fit data are calculated from a sum of the data for both approaching atoms, whereas volume data pertains to a single atom. The left-hand volume compression is for the X atom the right-hand for the hydrogen atom.

System	RMS Error (kJ/mol)	Energy Range (kJ/mol)	Min Energy (kJ/mol)	Max Energy (kJ/mol)	Coefficient A (kJ/mol)	Coefficient B (Å^{-1})	Change of Atomic volume (%)	
NH_3…NH_3	2.7	129.7	14.5	144.2	2,889	1.5734	-12.1	-38.5
OH_2…OH_2	2.5	86.6	14.3	100.9	1,213	1.3331	-5.8	-35.4
FH…FH	1.1	35.7	4.2	40.0	842	1.6591	-4.0	-47.0
PH_3…PH_3	0.8	82.4	3.8	86.2	2,641	1.6212	-16.9	-27.1
SH_2…SH_2	1.9	89.8	-2.0	87.8	22,879	2.6386	-7.0	-32.8
ClH…ClH	2.3	50.1	-2.6	47.5	137,460	3.7801	-5.7	-24.2
CH_4…NH_3	1.4	133.9	9.4	143.3	4,579	1.8071	-12.8	-32.8
CH_4…OH_2	1.3	111.2	7.1	118.3	4,457	1.9144	-6.6	-26.8
CH_4…FH	1.1	81.6	4.5	86.1	4,460	2.1216	-4.2	-23.1
NH_3…OH_2	2.5	109.0	11.8	120.8	2,627	1.6360	-6.1	-31.7
NH_3…FH	1.8	72.6	6.2	78.8	2,876	1.9434	-4.0	-27.5
OH_2…NH_3	2.7	103.4	17.2	120.6	1,435	1.3090	-11.6	-41.1
OH_2…FH	1.8	57.7	6.4	64.2	1,532	1.7248	-5.1	-30.9
FH…NH_3	1.9	57.9	15.5	73.4	478	1.0039	-11.2	-38.6
FH…OH_2	6.1	64.1	-12.0	52.1	21,428	3.1289	-5.5	-35.3

For all following figures, decomposed intra-atomic energy plots are displayed on the right and plots of the number of electrons are displayed on the left. The first line of plots gives data for the heavier atom, and the second line of plots gives data for the hydrogen atom.

2.1. Unmixed systems

2.1.1 $NH_3...NH_3$

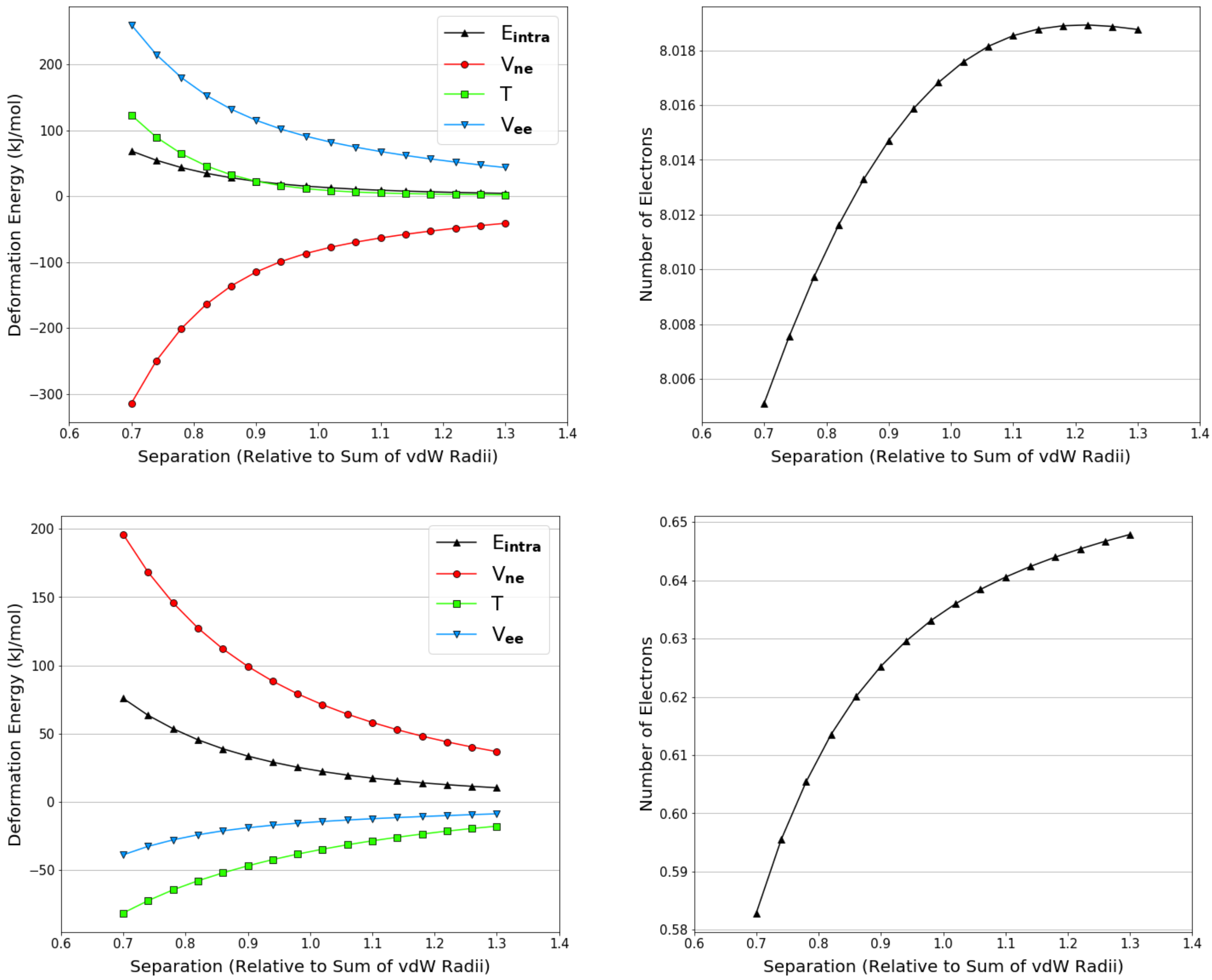

Figure S7. Intra-atomic energies and number of electrons for N and H atoms in the NH_3 dimer.

2.1.2 $OH_2...OH_2$

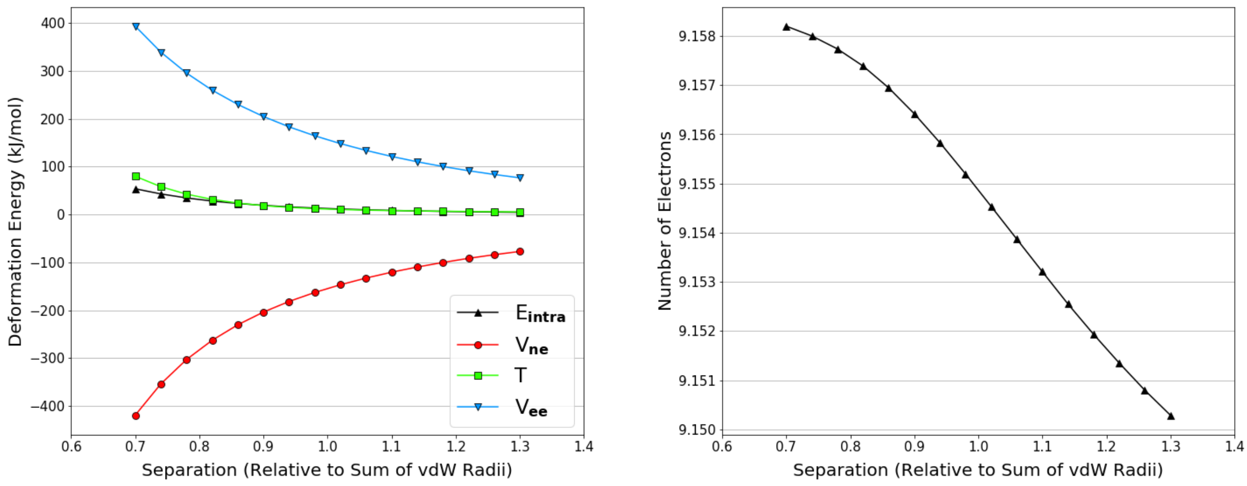

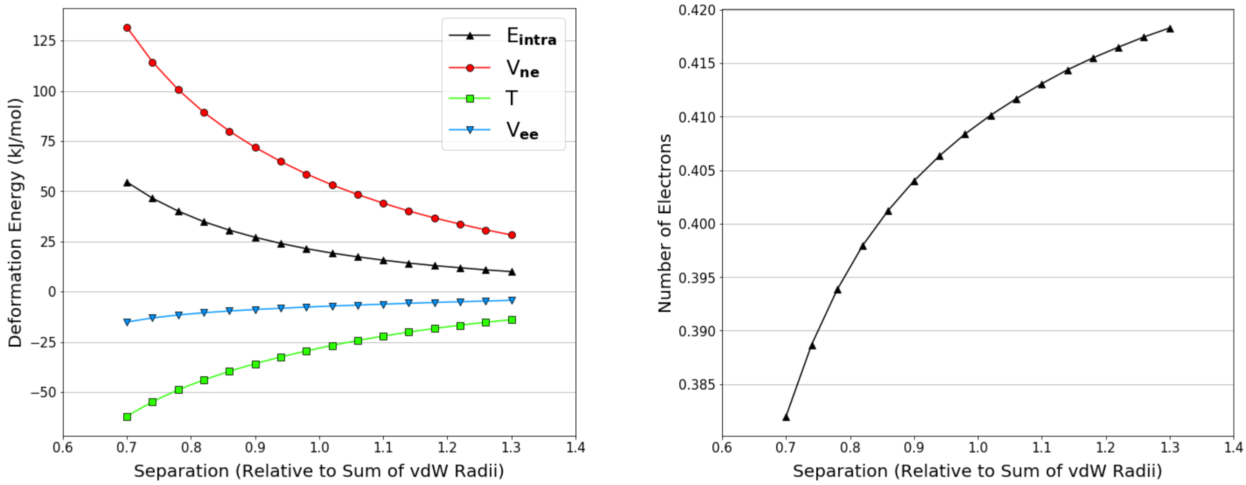

Figure S8. Intra-atomic energies and number of electrons for O and H atoms in the H_2O dimer.

2.1.3 FH…FH

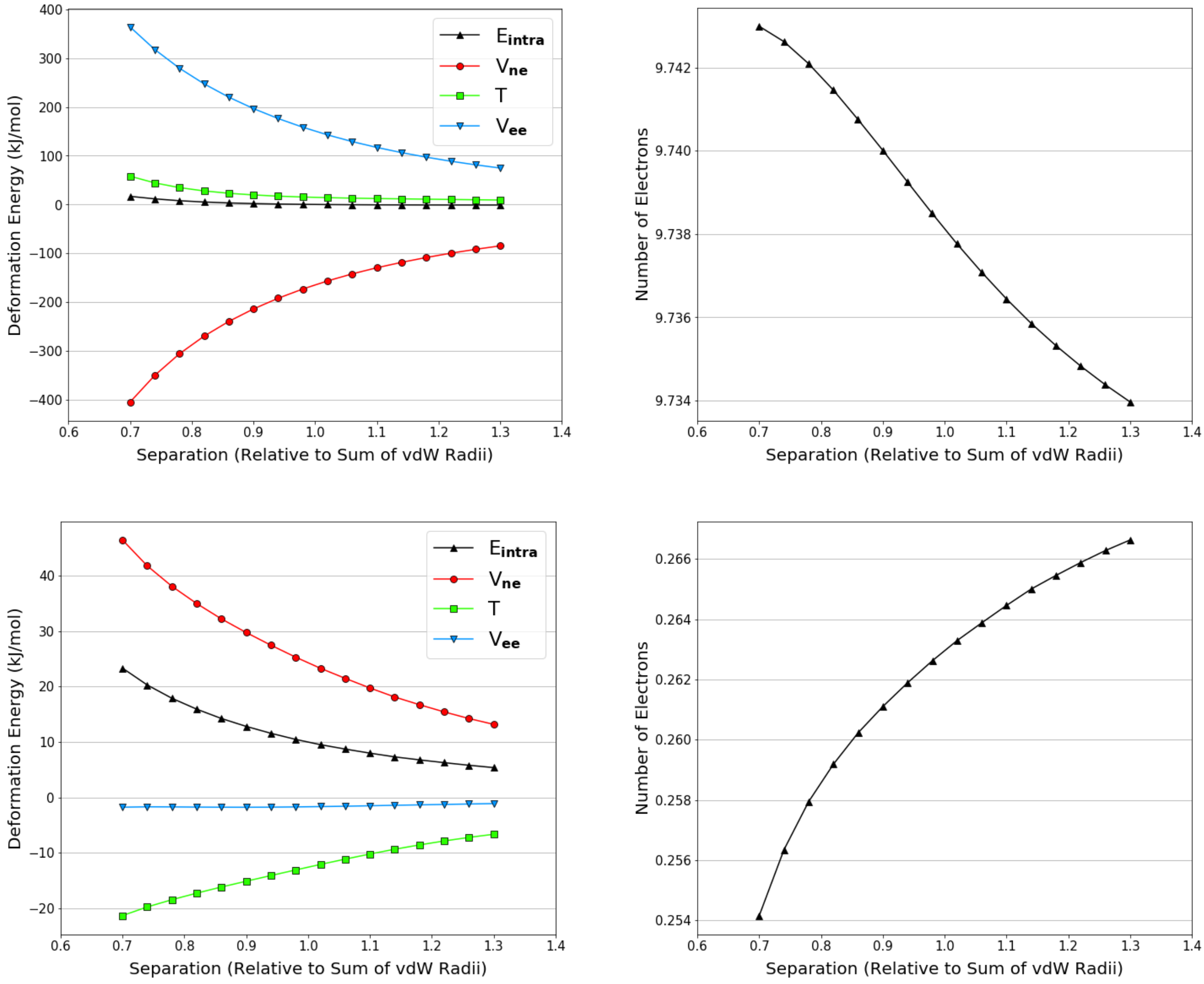

Figure S9. Intra-atomic energies and number of electrons for F and H atoms in the HF dimer.

2.1.4 $PH_3 \ldots PH_3$

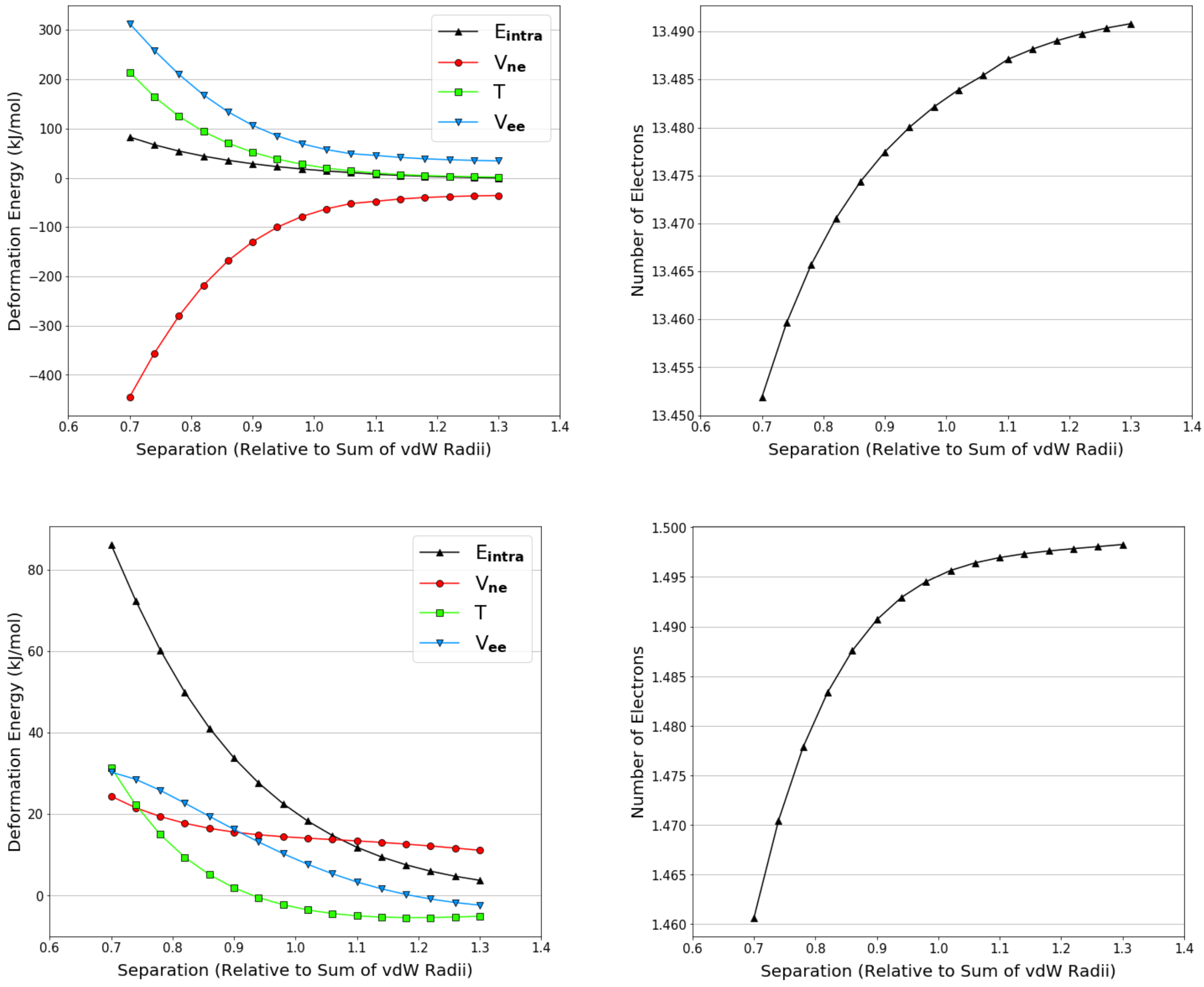

Figure S10. Intra-atomic energies and number of electrons for N and H atoms in the PH_3 dimer.

2.1.5 $SH_2 \ldots SH_2$

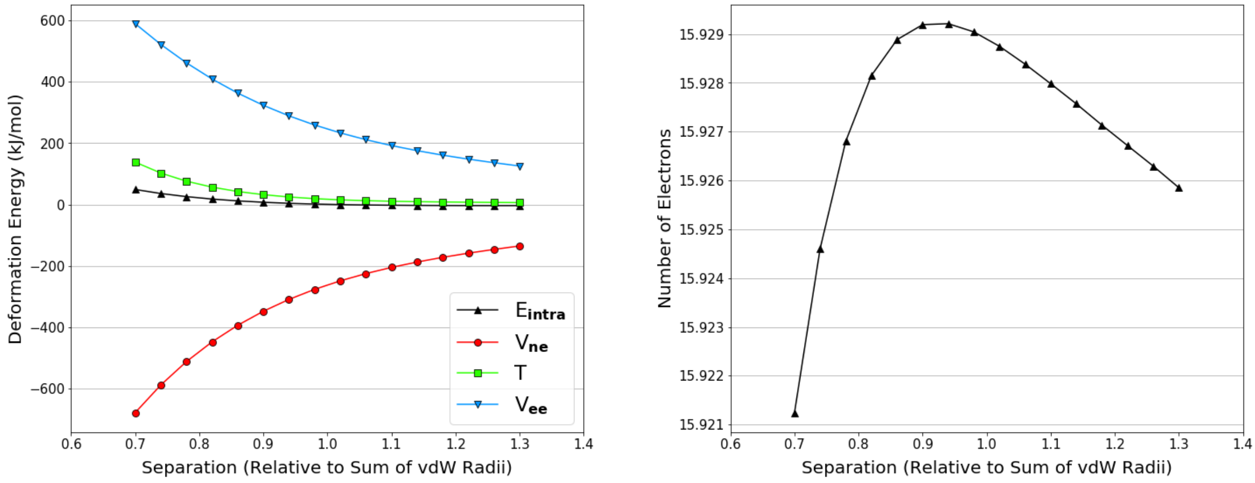

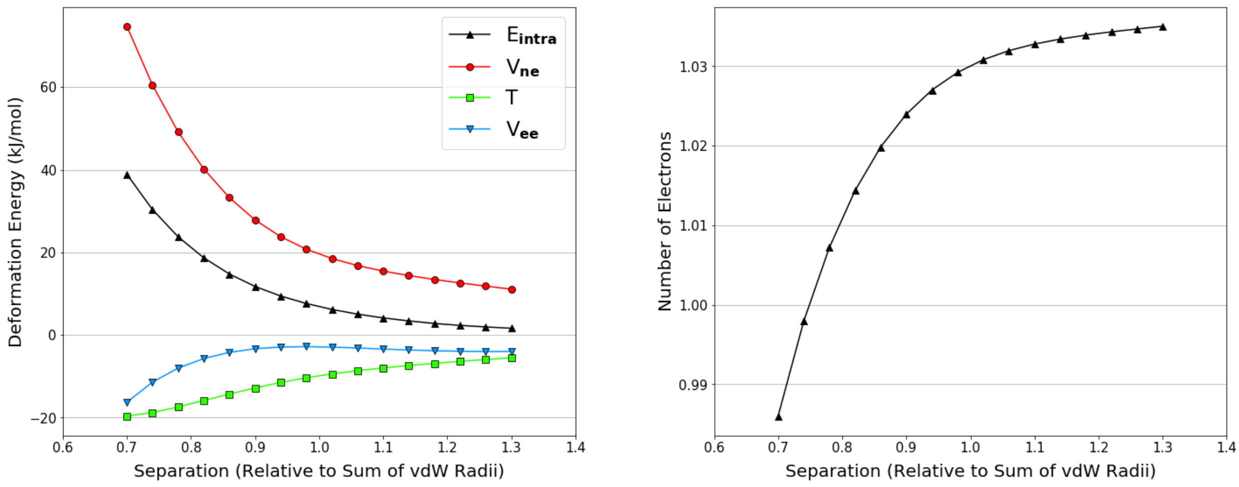

Figure S11. Intra-atomic energies and number of electrons for S and H atoms in the H_2S dimer.

2.1.6 ClH…ClH

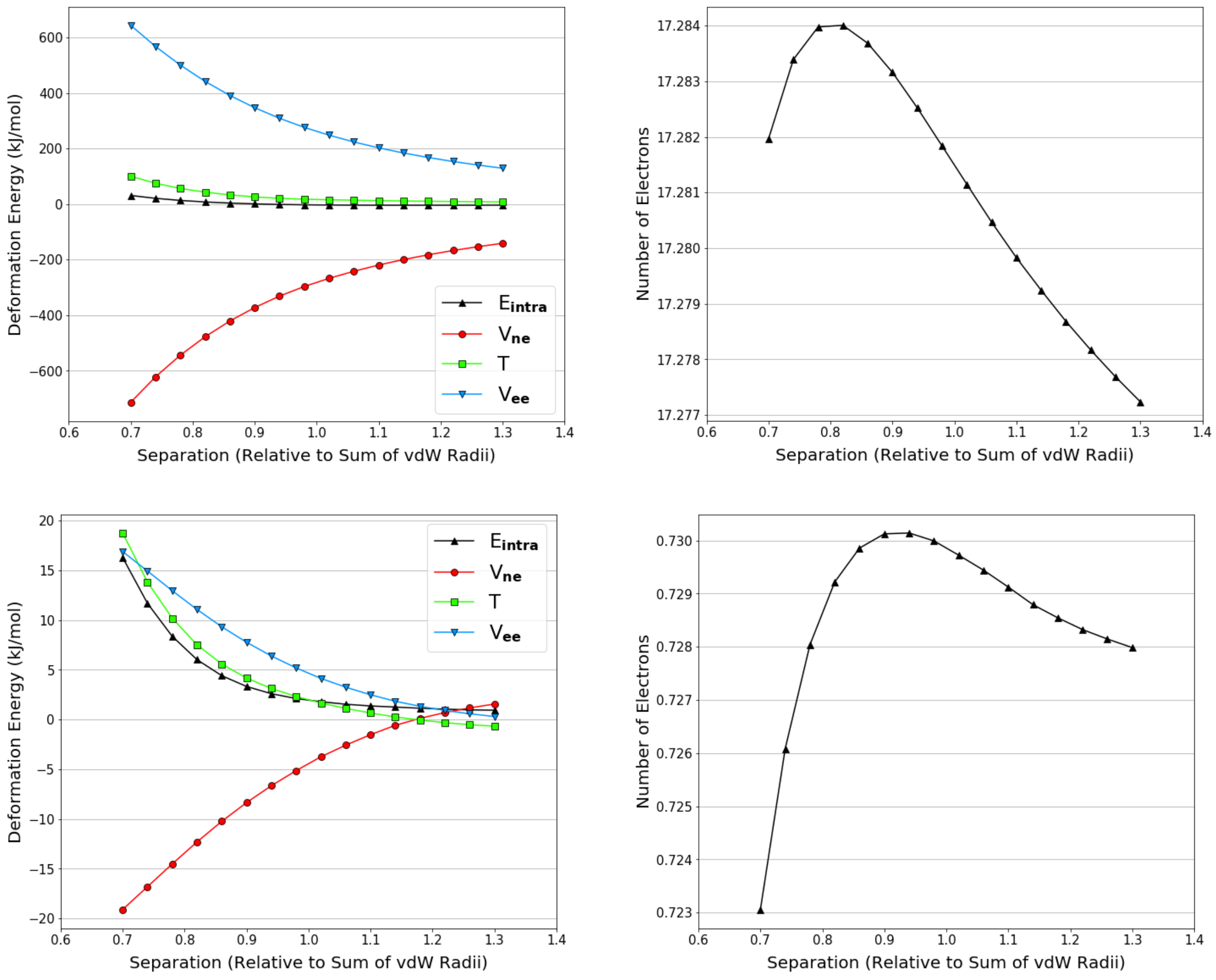

Figure S12. Intra-atomic energies and number of electrons for Cl and H atoms in the HCl dimer.

All intra-atomic energies for atoms in unmixed H…X approaches behave according to the model set out in the discussion. Most hydrogen atoms have intra-atomic energies dictated by the V_{ne} contribution, the exceptions being the PH_3 and HCl dimers. In the PH_3 dimer, the hydrogen atoms are negatively charged so have more electrons and as such are more affected by atomic compression, so the deformation energies show trends originating from a combination of charge and volume effects. In the HCl dimer, the change in the number of electrons during the approach is an order of magnitude smaller than in other systems; 0.007 electrons in the HCl dimer compared to 0.04 electrons in the H_2S dimer. Generally, the maximum deformation of the intra-atomic energies for both atoms decreases as atom X moves along the period, as is evident in the fit coefficients displayed in Table S7. This trend is not rigorous, however, as multiple variables change when X changes.

2.2. Mixed Systems

2.2.1 CH_4…NH_3

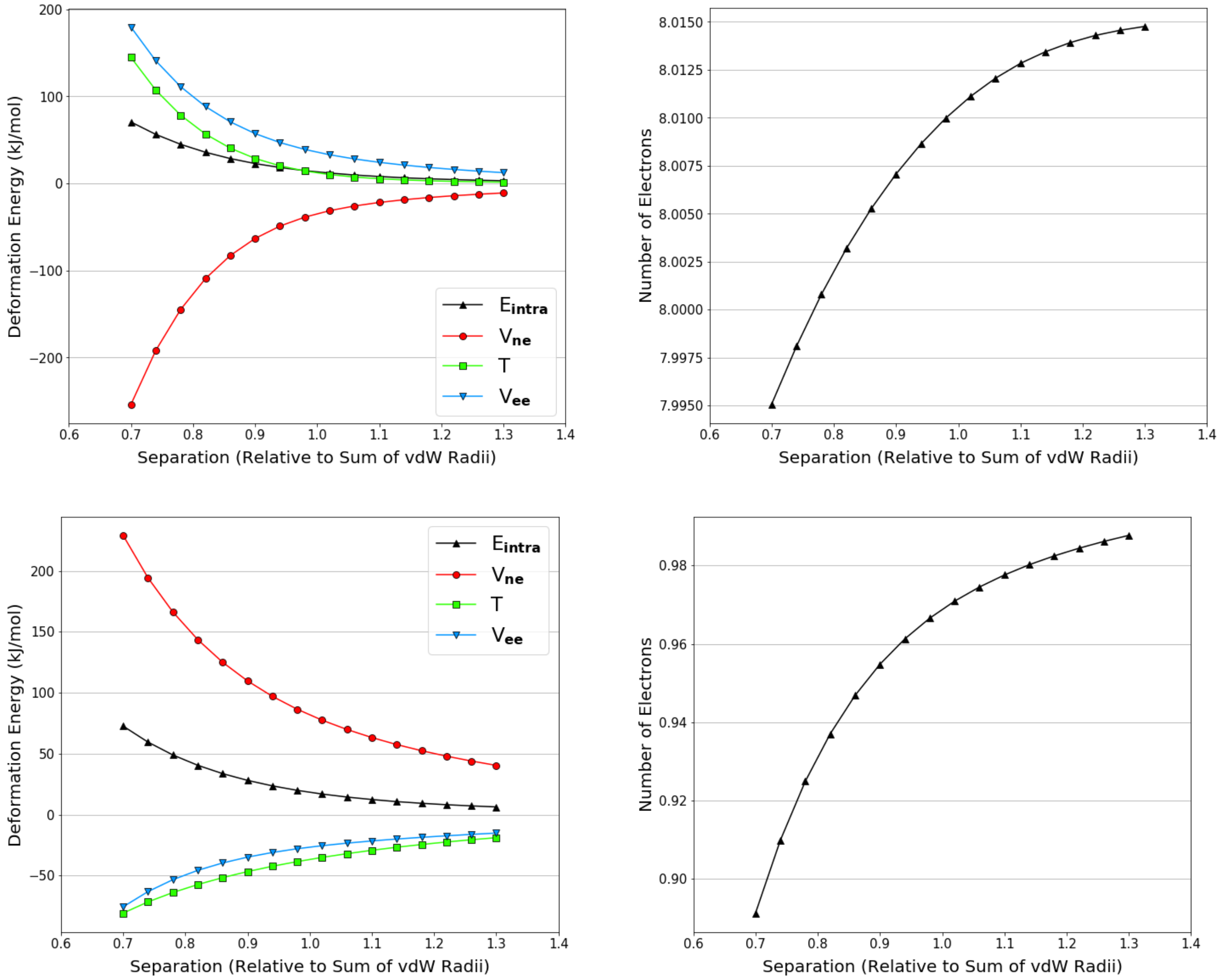

Figure S13. Intra-atomic energies and number of electrons for N and H atoms as a hydrogen atom in CH_4 approaches the nitrogen atom in NH_3.

2.2.2 CH_4...OH_2

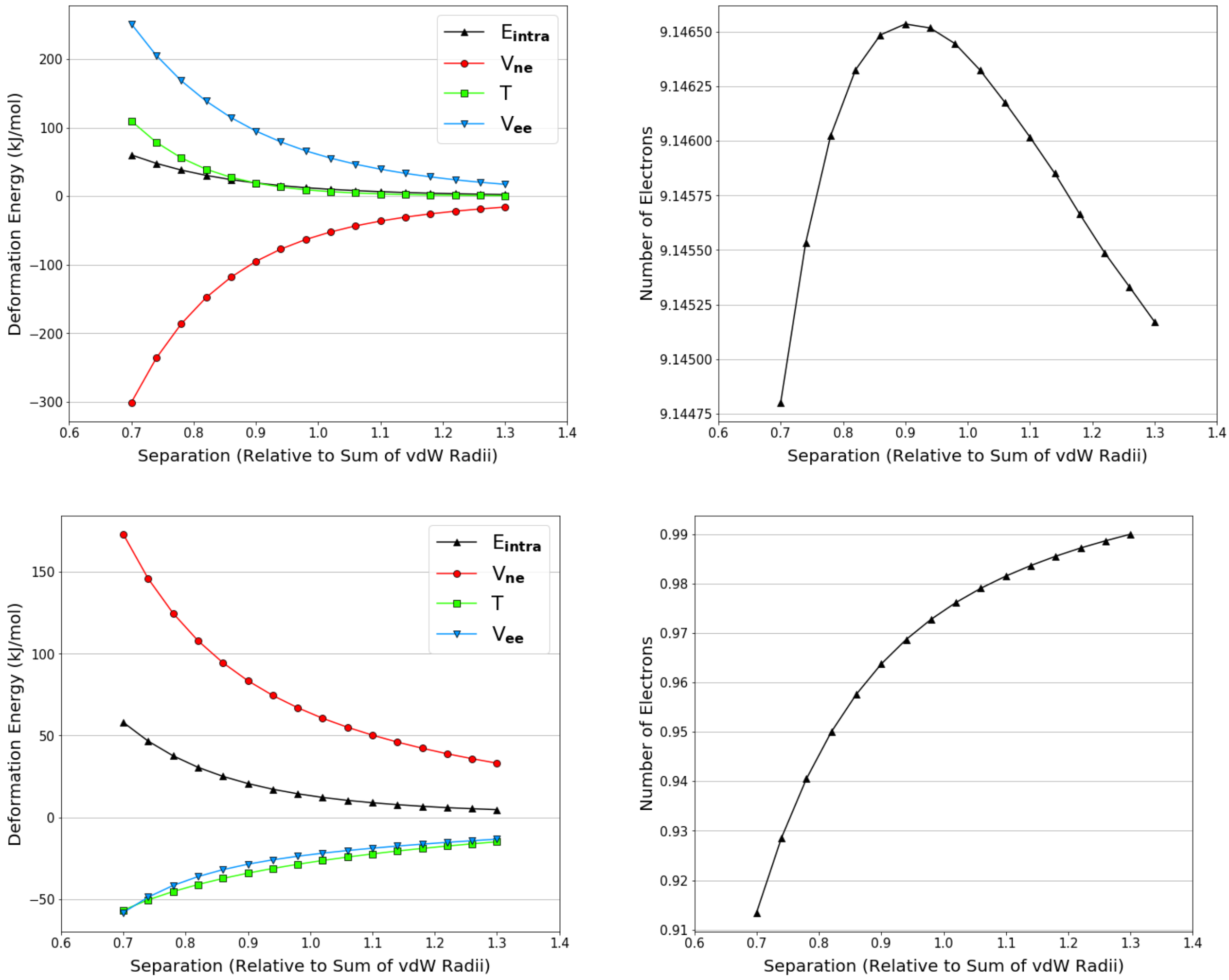

Figure S14. Intra-atomic energies and number of electrons for O and H atoms as a hydrogen atom in CH_4 approaches the oxygen atom in H_2O.

2.2.3 CH_4...FH

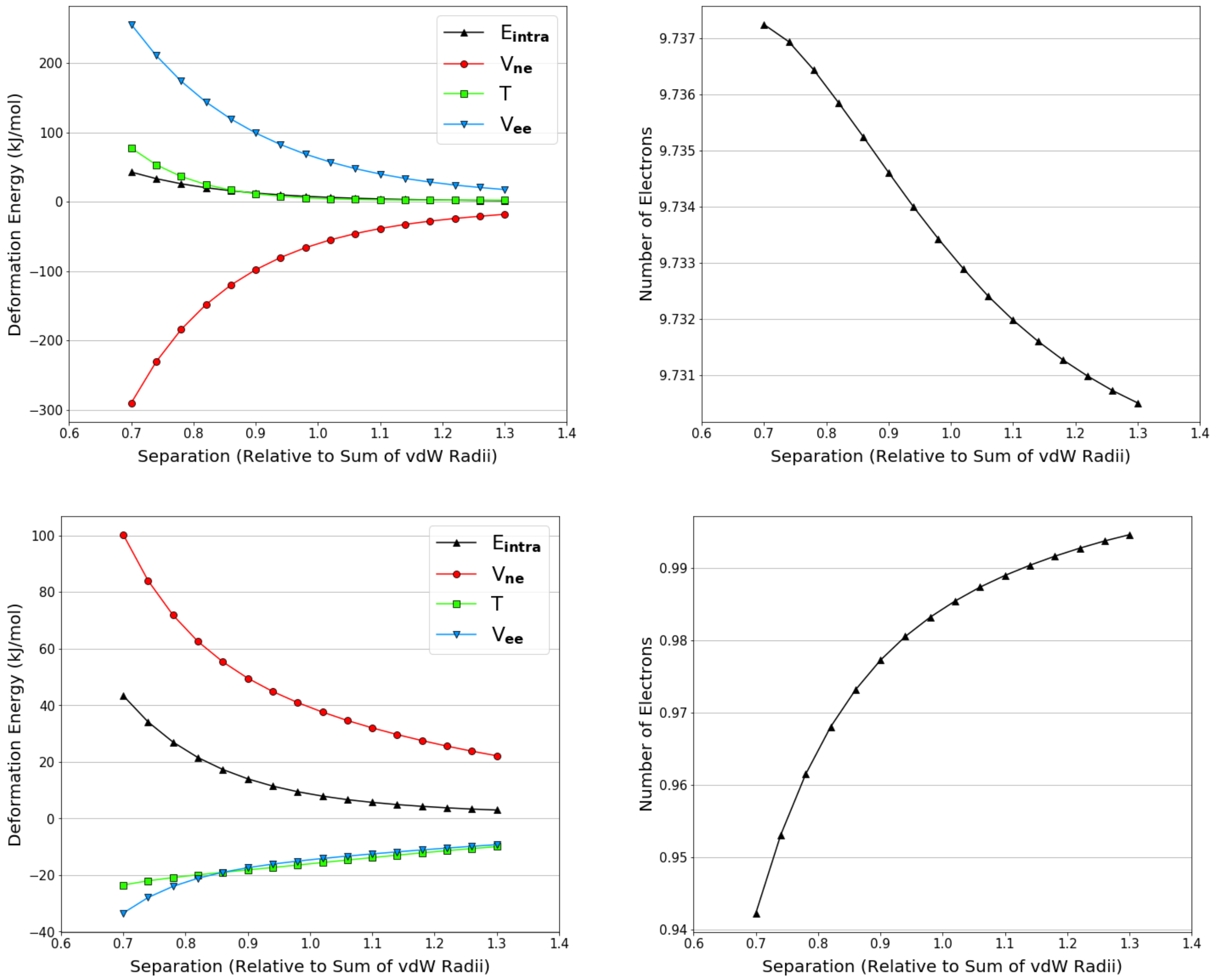

Figure S15. Intra-atomic energies and number of electrons for F and H atoms as a hydrogen atom in CH_4 approaches the fluorine atom in HF.

2.2.4 NH_3...OH_2

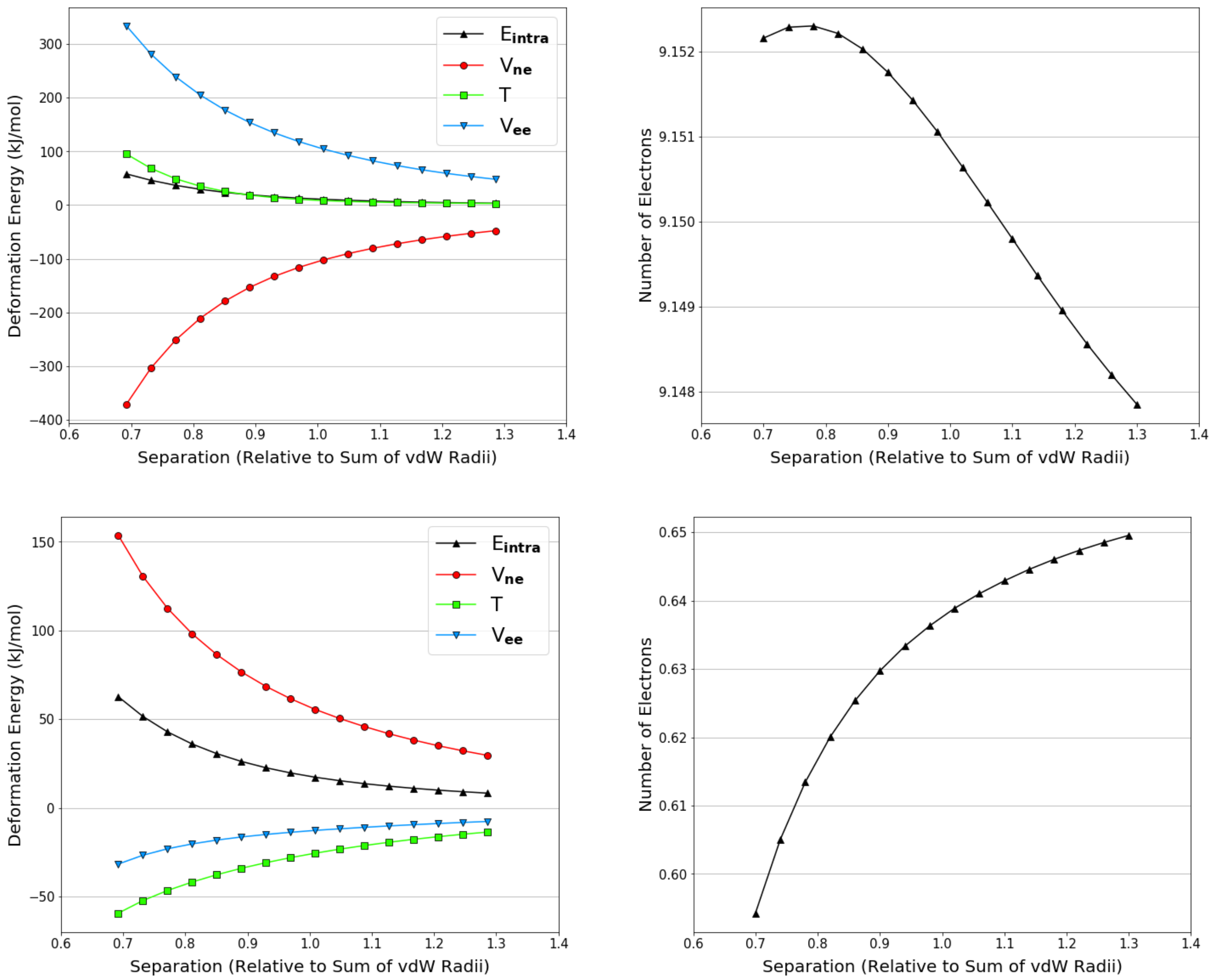

Figure S16. Intra-atomic energies and number of electrons for O and H atoms as a hydrogen atom in NH_3 approaches the oxygen atom in H_2O.

2.2.5 NH_3…FH

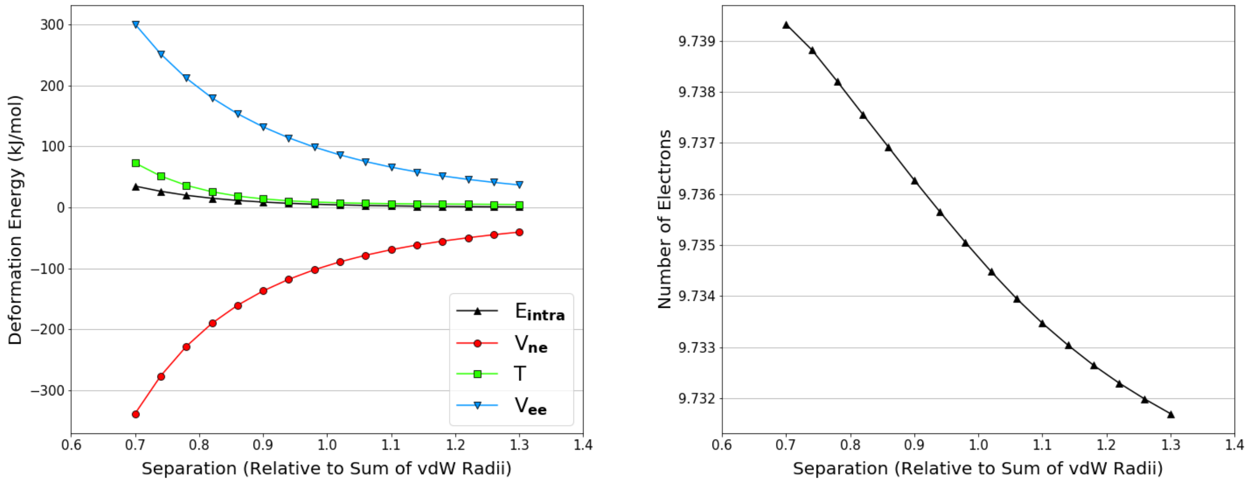

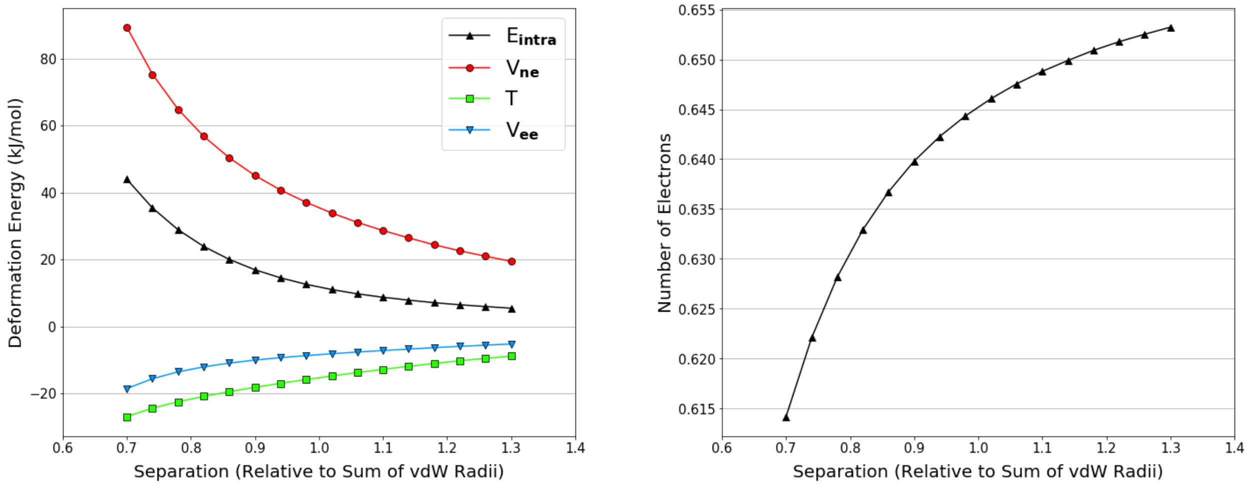

Figure S17. Intra-atomic energies and number of electrons for F and H atoms as a hydrogen atom in NH_3 approaches the fluorine atom in HF.

2.2.6 $OH_2 \ldots NH_3$

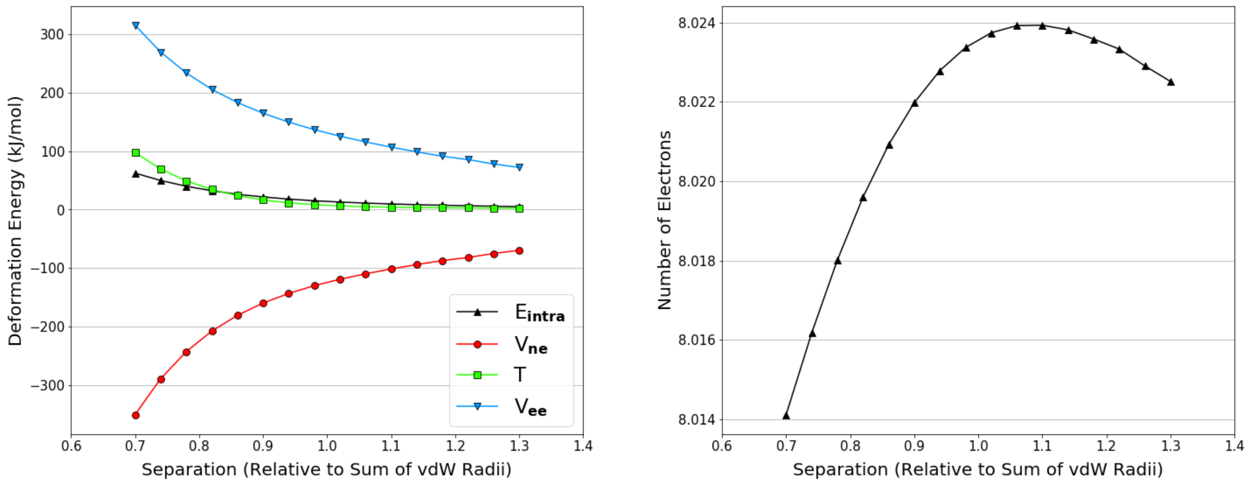

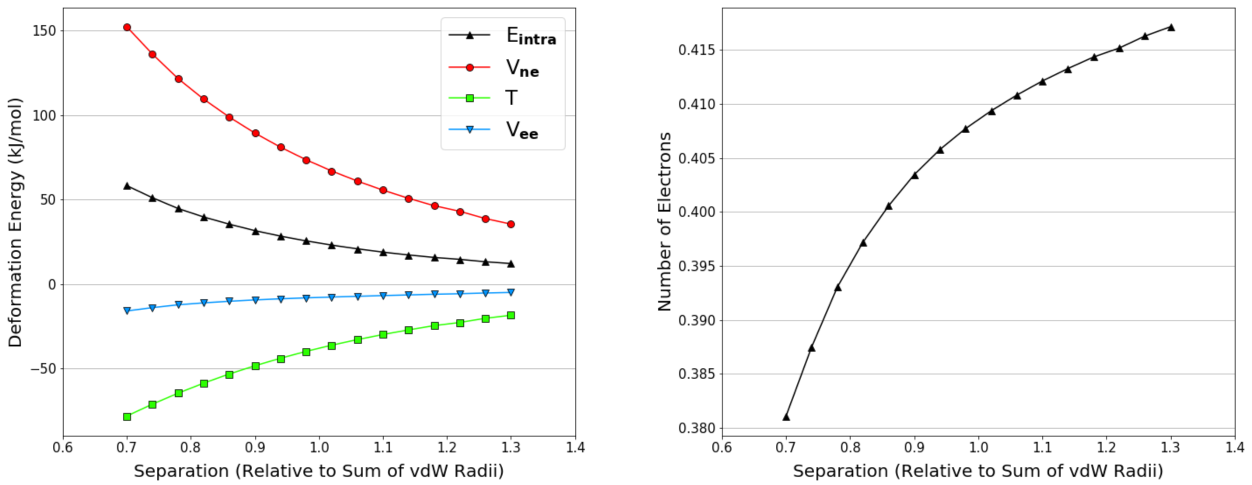

Figure S18. Intra-atomic energies and number of electrons for N and H atoms as a hydrogen atom in H_2O approaches the nitrogen atom in NH_3.

2.2.7. OH_2...FH

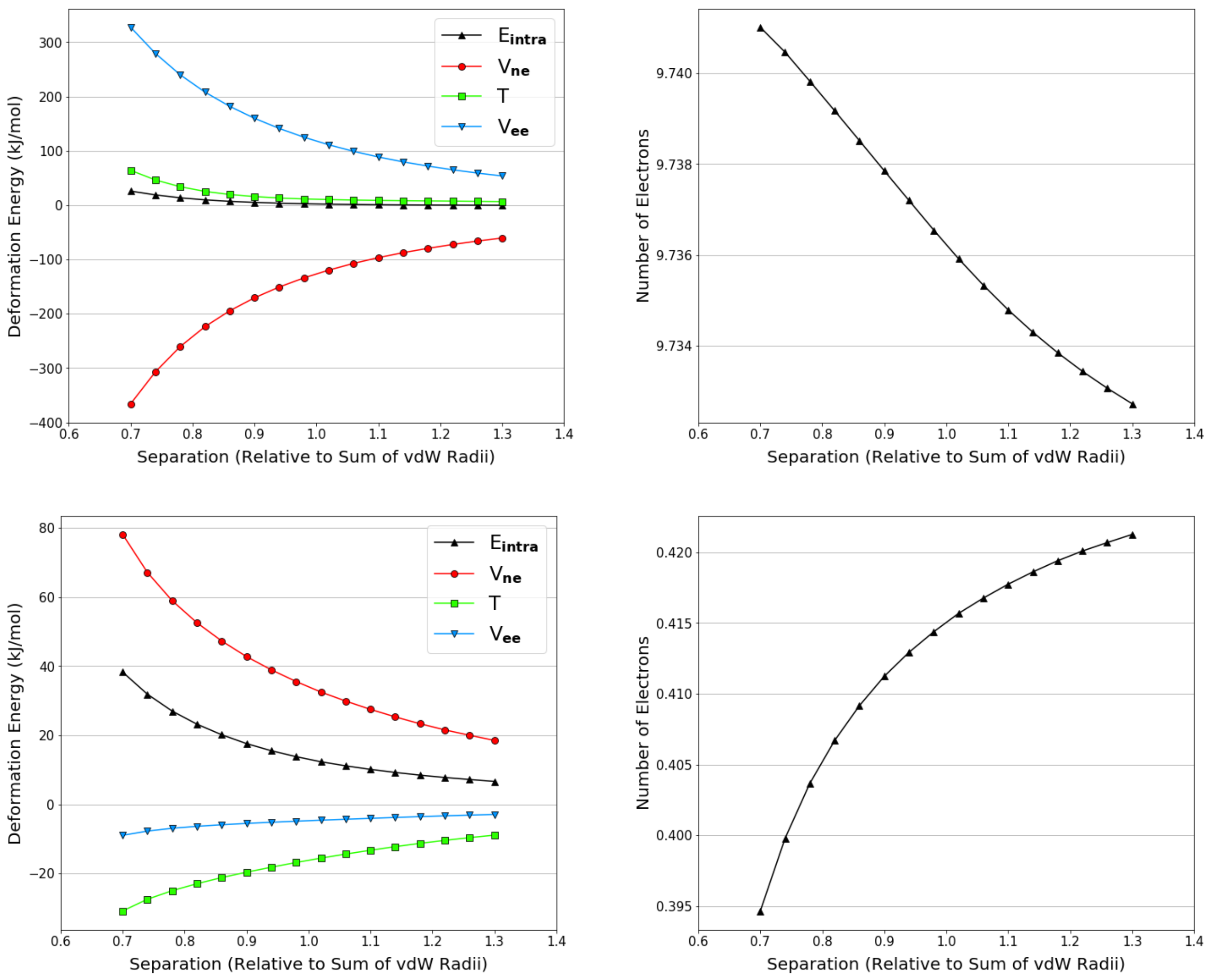

Figure S19. Intra-atomic energies and number of electrons for F and H atoms as a hydrogen atom in H_2O approaches the nitrogen atom in HF.

2.2.8. FH…NH_3

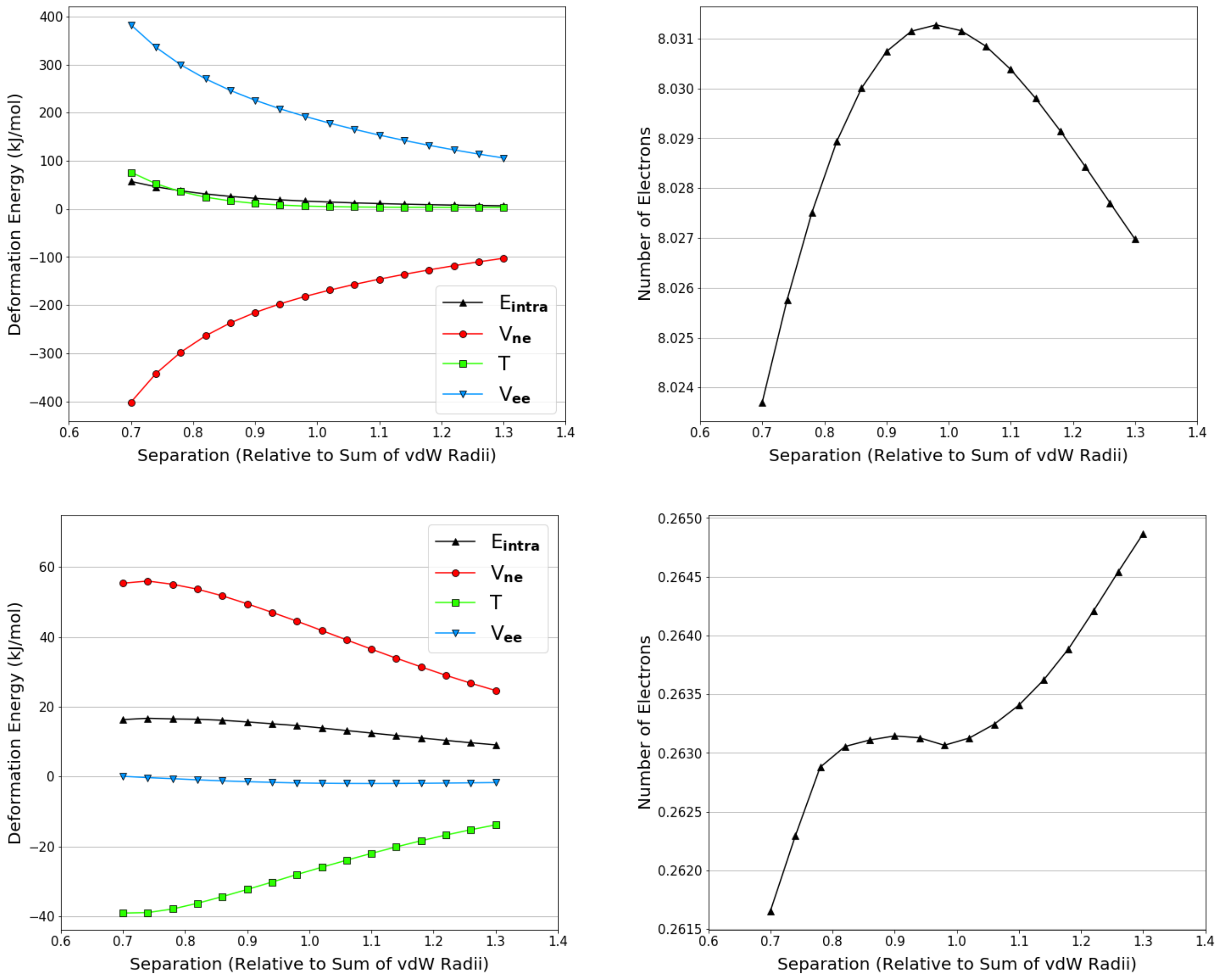

Figure S20. Intra-atomic energies and number of electrons for N and H atoms as the hydrogen atom in HF approaches the nitrogen atom in NH_3.

2.2.9. FH…OH_2

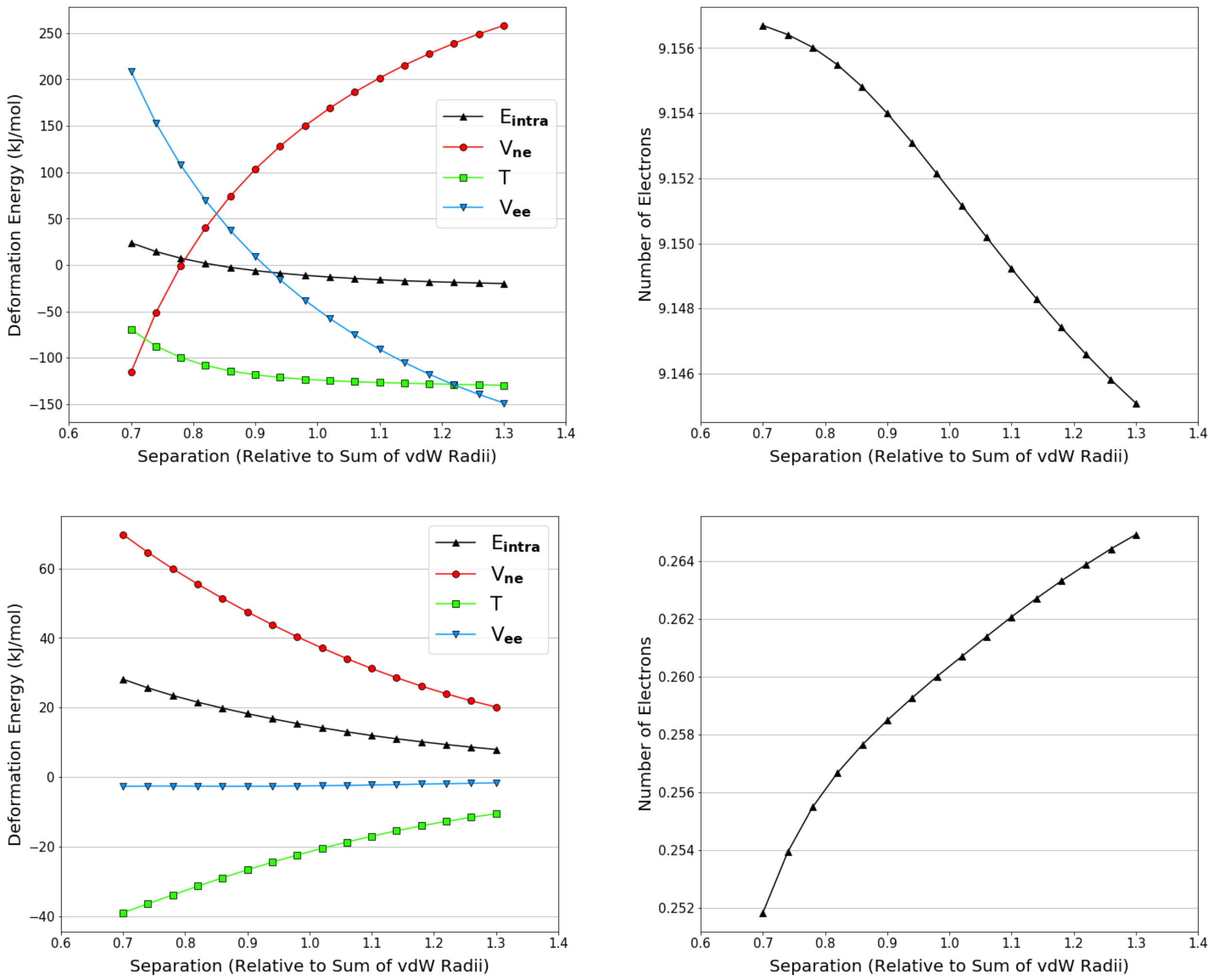

Figure S21. Intra-atomic energies and number of electrons for O and H atoms as the hydrogen atom in HF approaches the oxygen atom in H_2O.

The intra-atomic energies for mixed H…X approaches also behave according to the model set out in the discussion. Here, the periodic trends are easier to identify as only one molecule is changed at a time. For example, for the series CH_4…NH_3 to CH_4…FH, the maximum deformation of intra-atomic energy for both atoms decreases as atom X increases in mass. For the series CH_4…NH_3, NH_3…NH_3, OH_2…NH_3, FH…NH_3, the maximum deformation of intra-atomic energy for both atoms also decreases.

3. Hydrogen-hydrogen approaches (H…H)

3.1. Unmixed systems

3.1.1 $CH_4...H_4C$

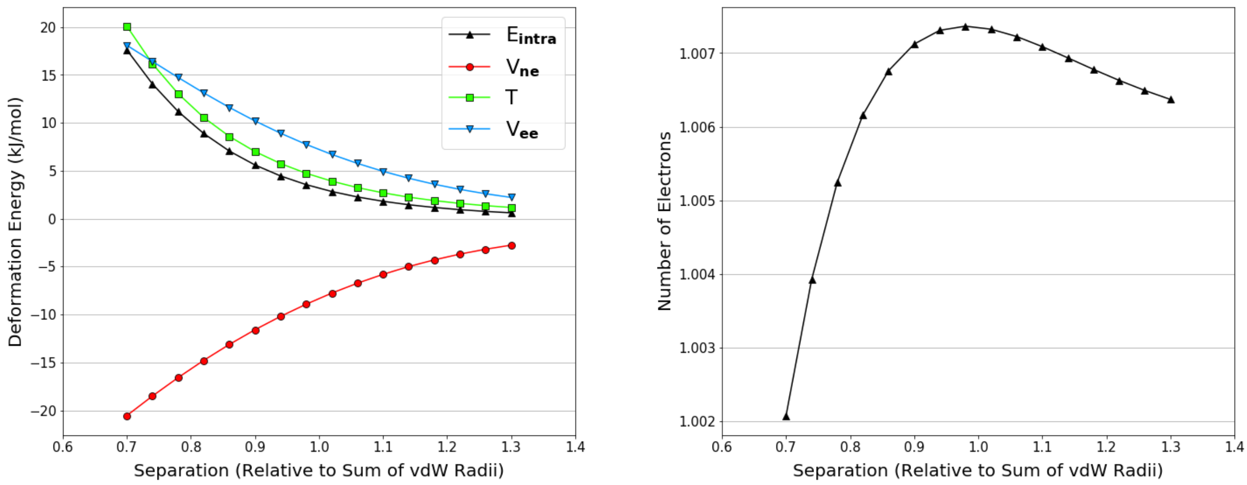

Figure S22. Intra-atomic energies and number of electrons for a single H atom as it approaches its mirror in the CH_4 dimer.

3.1.2 $NH_3...H_3N$

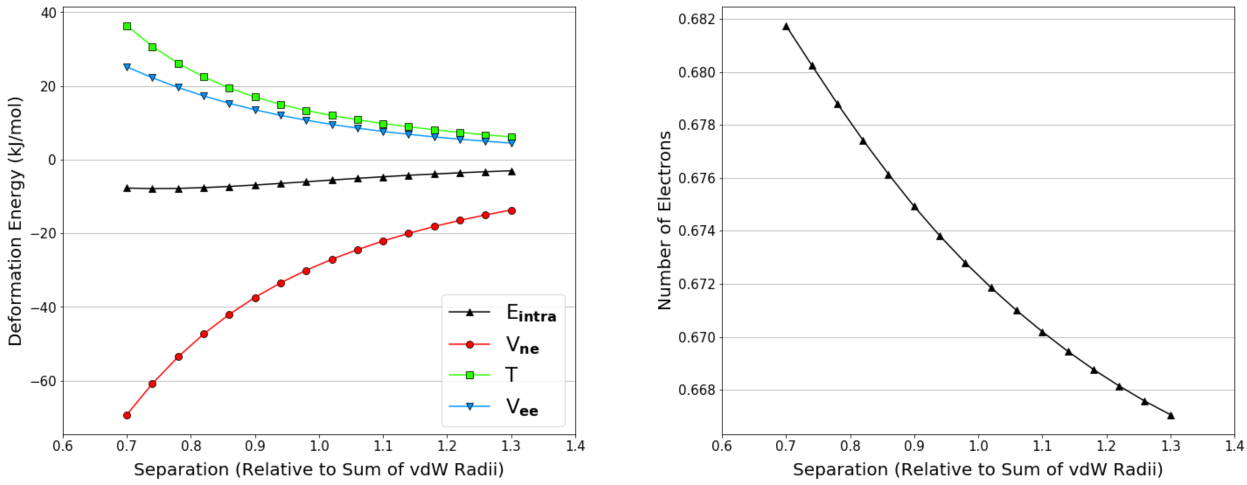

Figure S23. Intra-atomic energies and number of electrons for a single H atom as it approaches its mirror in the NH_3 dimer.

3.1.3 $OH_2...H_2O$

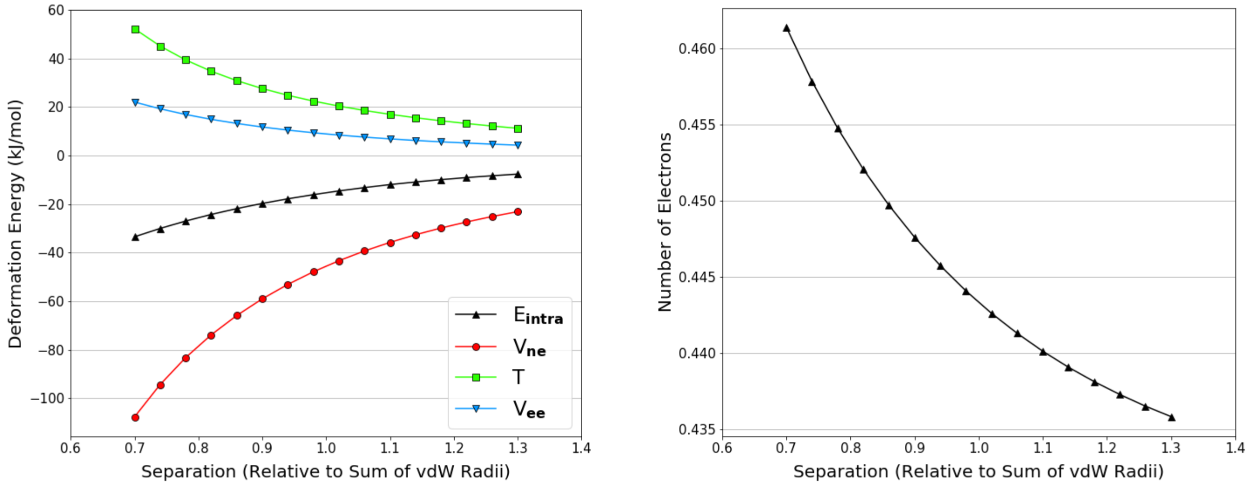

Figure S24. Intra-atomic energies and number of electrons for a single H atom as it approaches its mirror in the H_2O dimer.

<u>3.1.4 FH…HF</u>

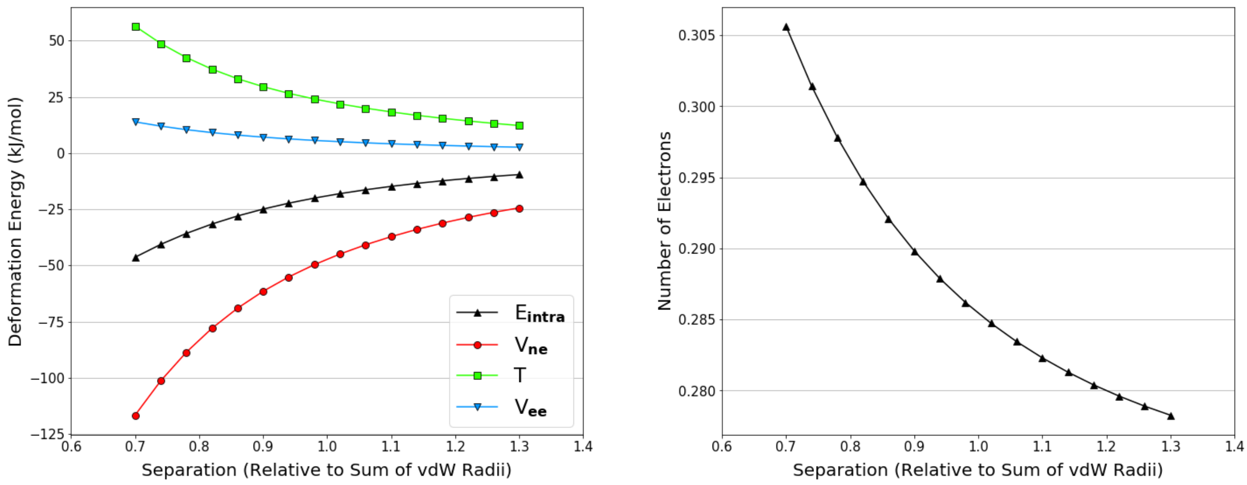

Figure S25. Intra-atomic energies and number of electrons for a single H atom as it approaches its mirror in the HF dimer.

These experiments are commented on at length in the discussion. Hydrogen atoms in CH_4 are essentially neutral and experience little change in the number of electrons as one hydrogen atom approaches another, so the deformation in its intra-atomic energy is determined by the T contribution and is therefore exponentially positive. In the other experiments, the hydrogen atoms are charged and experience significant increases the number of electrons as they approach each other. This leads to dominance of the V_{ne} contribution which is greater in magnitude than both the T and V_{ee} contributions and increasingly negative deformation in intra-atomic energy. The more positive the charge on the hydrogen atom, the more negative the intra-atomic deformation energy.

3.2. Mixed systems

For all following figures, data for the first hydrogen atom is displayed in the top row, and data for the second hydrogen atom is displayed in the second row. For example, in the CH_4…NH_3 approach, data for the hydrogen atom in methane is presented first, then data for the hydrogen atom in ammonia is presented second.

3.2.1. CH_4…H_3N

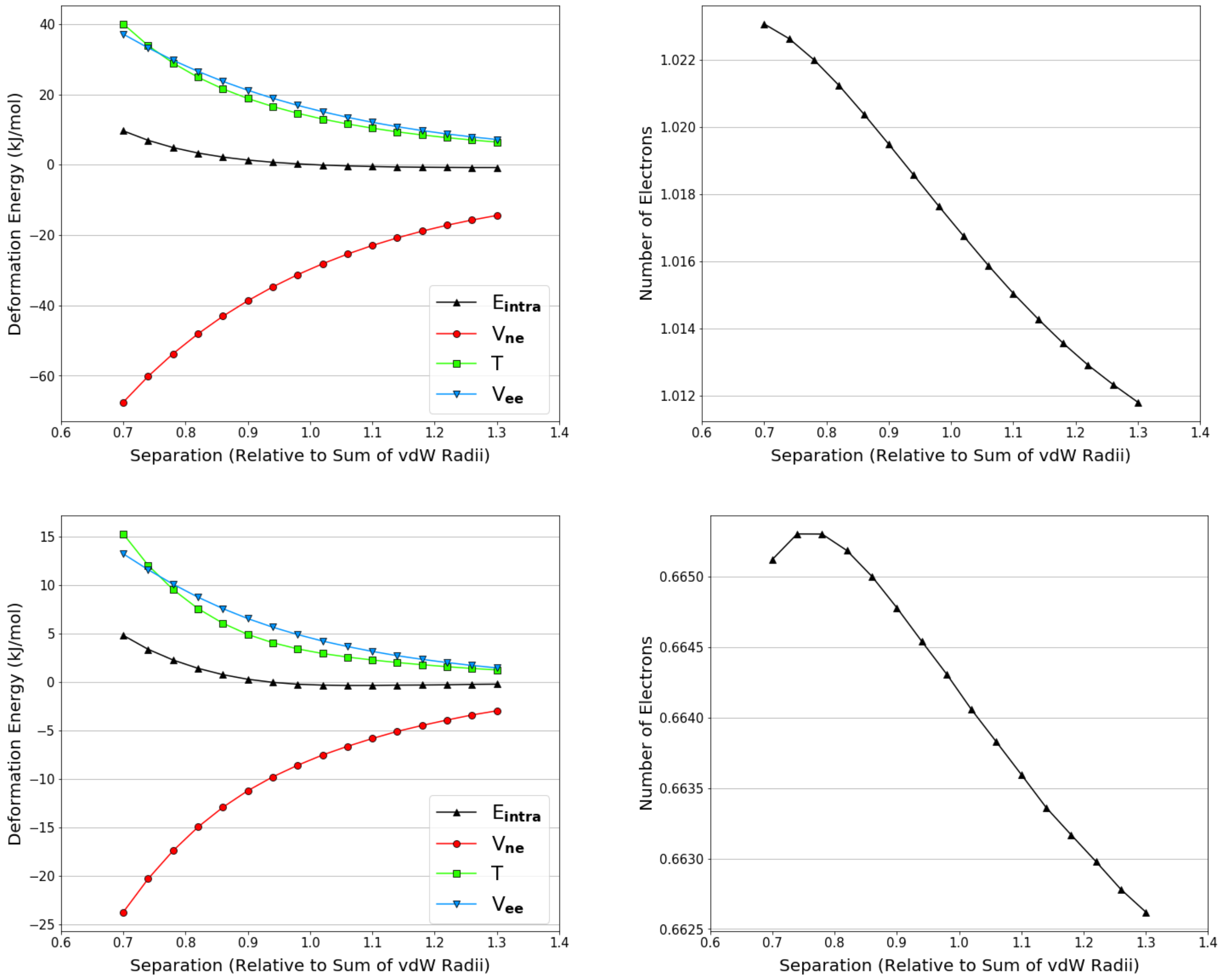

Figure S26. Intra-atomic energies and number of electrons for the hydrogen atom in CH_4 (top) and the hydrogen atom in NH_3 (bottom) as they approach each other.

3.2.2 $CH_4 \ldots H_2O$

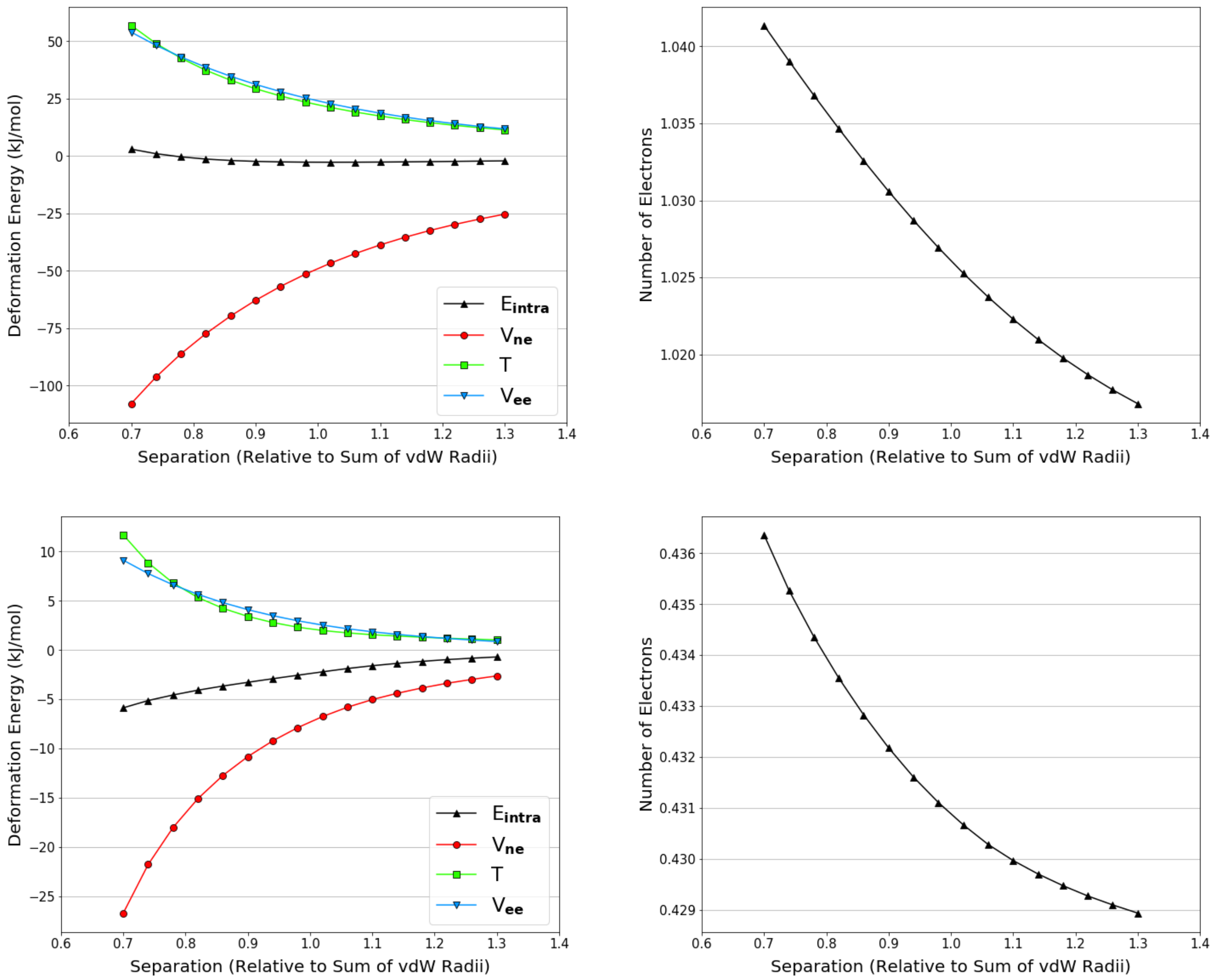

Figure S27. Intra-atomic energies and number of electrons for the hydrogen atom in CH_4 (top) and the hydrogen atom in H_2O (bottom) as they approach each other.

3.2.3 $CH_4 \ldots HF$

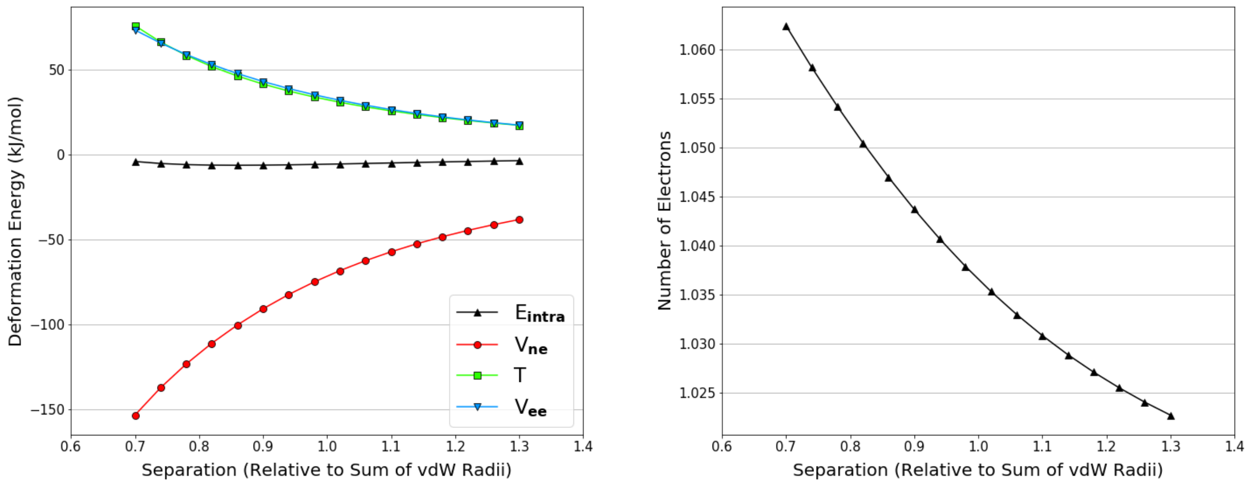

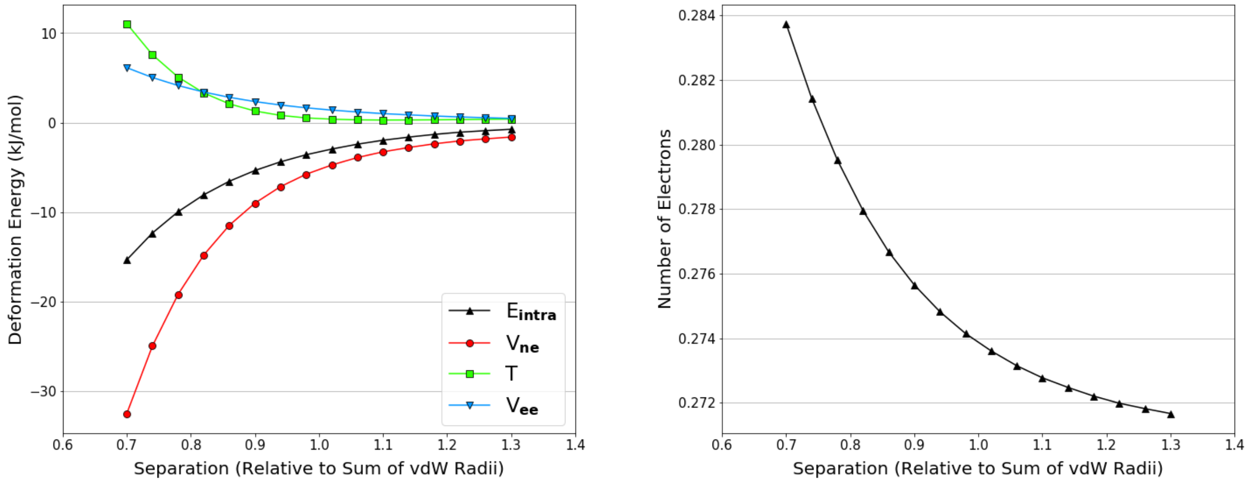

Figure S28. Intra-atomic energies and number of electrons for the hydrogen atom in CH_4 (top) and the hydrogen atom in HF (bottom) as they approach each other.

3.2.4. NH_3...H_2O

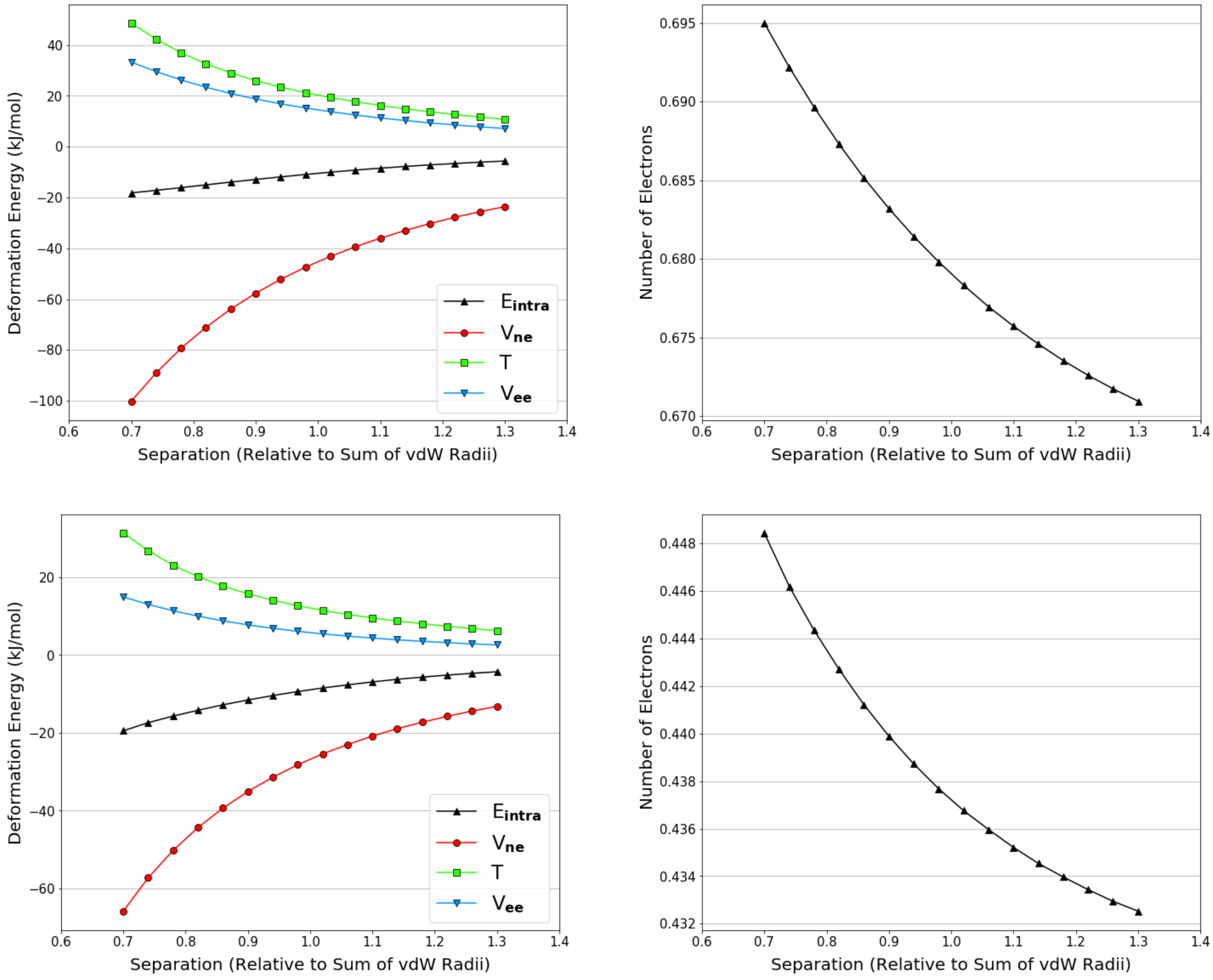

Figure S29. Intra-atomic energies and number of electrons for the hydrogen atom in NH_3 (top) and the hydrogen atom in H_2O (bottom) as they approach each other.

3.2.5 NH_3…HF

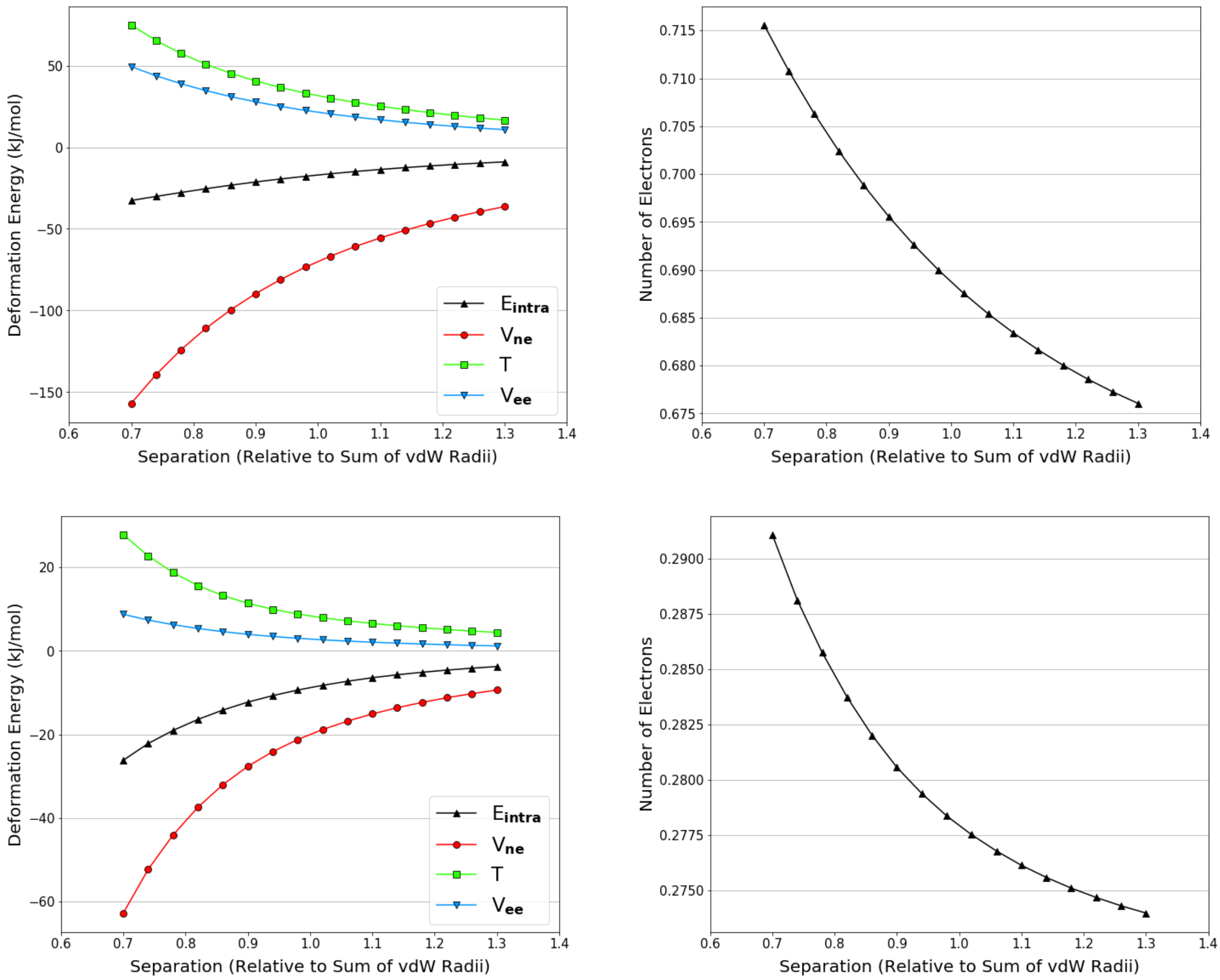

Figure S30. Intra-atomic energies and number of electrons for the hydrogen atom in NH_3 (top) and the hydrogen atom in HF (bottom) as they approach each other.

3.2.6. OH_2...HF

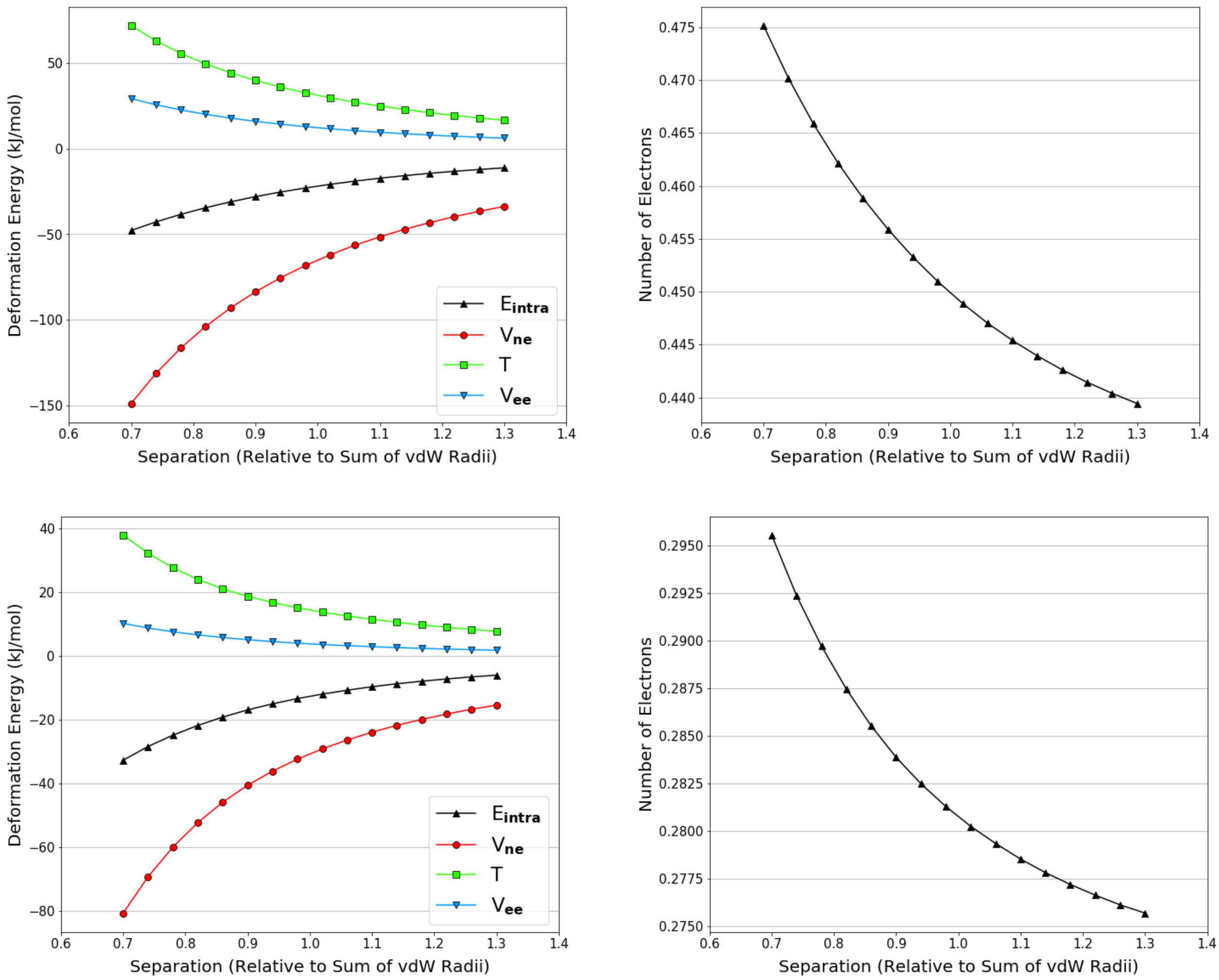

Figure S31. Intra-atomic energies and number of electrons for the hydrogen atom in H_2O (top) and the hydrogen atom in HF (bottom) as they approach each other.

The data from the mixed H...H approaches is consistent with the model outlined in the discussion. All atoms experience comparable increases in electrons as they approach their counterpart, and the extent to which the deformation in intra-atomic energy is determined by the T contribution or the V_{ne} contribution depends on the charge of the atom. Highly charged atoms have few electrons to be affected by an atomic compression but are equally influenced by changes to electron population so are proportionately more dependent on charge deformations.

4. Relaxed experiments

The intra-atomic energy data for rigid experiments were subtracted from the data for corresponding relaxed experiments to calculate the deviation between them.

4.1 HF theta scan

Table S8. Energy ranges and exponential fit data for the relaxed HF theta scan. Energy ranges and fit data are calculated from a sum of the data for both approaching atoms, whereas volume data pertains to a single atom. The fit data in Table S8 follow very similar trends to the rigid scan in Table S3.

Theta	RMS Error (kJ/mol)	Energy Range (kJ/mol)	Minimum Energy (kJ/mol)	Maximum Energy (kJ/mol)	Coefficient A (kJ/mol)	Coefficient B (Å^{-1})	Change of Atomic Volume (%)
0	2.0	146.4	4.5	151.0	20,865	2.4032	-7.6
10	2.0	147.9	4.5	152.4	21,457	2.4122	-7.7
20	1.9	152.2	4.3	156.5	23,024	2.4332	-7.8
30	1.8	159.1	4.1	163.2	25,133	2.4550	-8.0
40	1.6	167.8	3.8	171.6	27,412	2.4718	-8.1
50	1.4	177.3	3.5	180.7	29,797	2.4862	-8.5
60	1.1	186.3	3.0	189.3	32,641	2.5072	-8.7
70	0.8	193.8	2.4	196.8	36,248	2.5393	-9.0
80	0.6	200.1	1.8	201.9	40,905	2.5831	-9.3
90	0.5	206.6	1.4	208.0	46,005	2.6251	-9.7
100	0.7	216.5	0.8	217.4	52,593	1.6675	-10.2
110	0.5	219.3	1.3	220.5	46,332	2.5958	-10.6
120	0.2	232.9	2.6	235.5	38,520	2.4769	-10.9
130	0.9	250.7	5.0	255.7	28,130	2.2805	-11.0
140	3.0	272.1	7.8	279.9	20,617	2.0775	-10.5
150	7.9	295.3	9.5	304.8	19,219	1.9844	-10.0
160	4.9	217.6	9.4	227.0	10,192	1.8375	-5.0
170	7.3	186.4	8.6	195.0	313,946	3.2390	-5.6

180	20,559	35.8	7.6	43.4	837,408	1.2805	-7.2

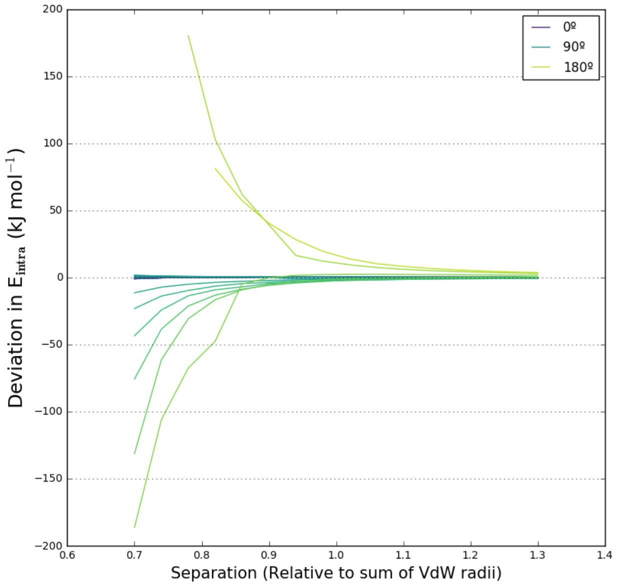

Figure S32. Gradient plot showing the deviation of the relaxed experiment from the rigid experiment.

<u>4.2 NH_3...NH_3</u>

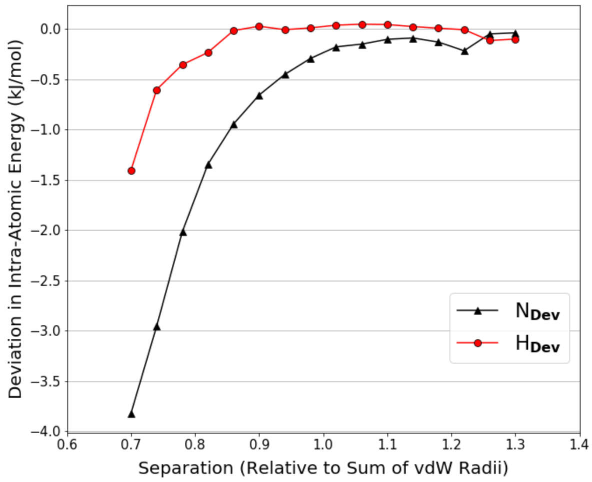

Figure S33. Deviations in the intra-atomic energies of approaching hydrogen and nitrogen atoms between relaxed and rigid NH_3 molecules.

Table S8. Energy ranges and exponential fit data for this approach. Fits were calculated for the sums of the intra-atomic energies of the approaching atoms.

**System**	**RMS Error**	**Energy Range (kJ/mol)**	**Min Energy (kJ/mol)**	**Max Energy (kJ/mol)**	**Coefficient A (kJ/mol)**	**Coefficient B (Å^{-1})**
NH_3...NH_3	2.4	124.6	14.4	139.0	2,604	1.5376

4.3 $NH_3 \ldots H_3N$

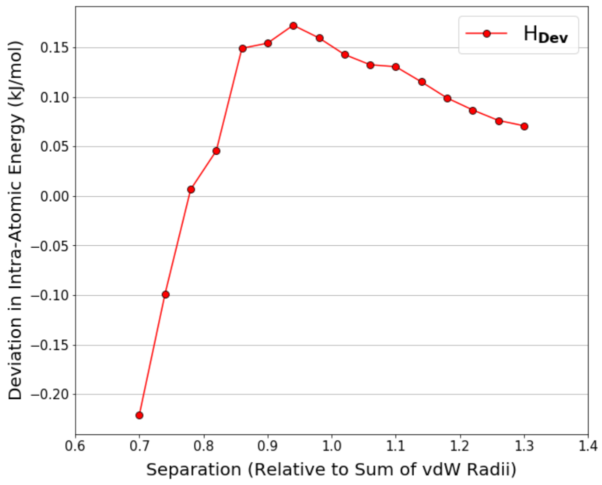

Figure S34. Deviation in the intra-atomic energy of an approaching hydrogen atom between relaxed and rigid NH_3 molecules.

4.4 $OH_2 \ldots OH_2$

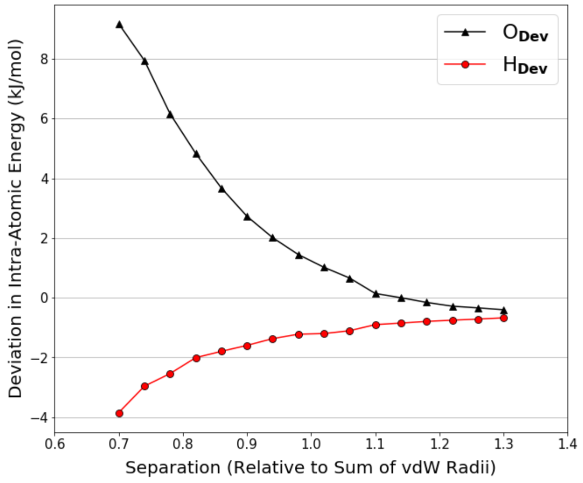

Figure S35. Deviations in the intra-atomic energies of approaching hydrogen and oxygen atoms between relaxed and rigid H_2O molecules.

Table S9. Energy ranges and exponential fit data for this approach. Fits were calculated for the sums of the intra-atomic energies of the approaching atoms.

System	RMS Error	Energy Range (kJ/mol)	Min Energy (kJ/mol)	Max Energy (kJ/mol)	Coefficient A (kJ/mol)	Coefficient B (Å^{-1})
H_2O-H_2O	2.2	93.1	13.2	106.3	1,521	1.4180

4.5 OH_2...H_2O

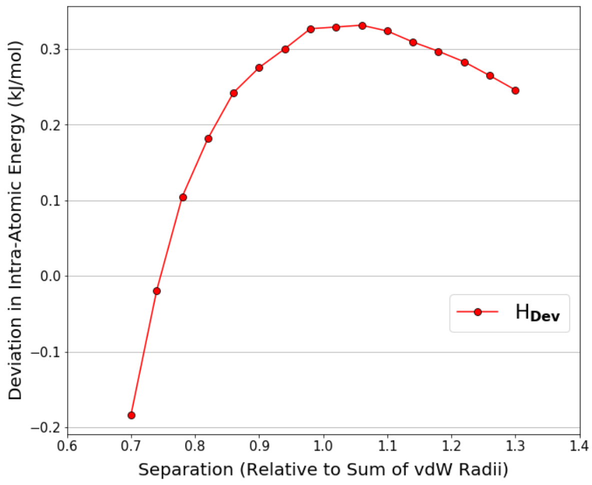

Figure S36. Deviation in the intra-atomic energy of an approaching hydrogen atom between relaxed and rigid H_2O molecules.

The deviations increase as separation decreases, and the relaxed systems are generally lower energy than the rigid ones, however this is not the case for the oxygen atom in the OH_2...OH_2 approach. Although one might always expect the intra-atomic energy to be lower in a relaxed system than a rigid one, this is only one component of the total energy that is minimised during geometry optimisation so any increases in intra-atomic energy upon relaxation will be offset by reductions in other energies.

5. Set of example coordinates

We provide a set of Cartesian coordinates for one of the experiments as an example to aid anyone that desires to reproduce any of the experiments. The experiment given is the linear FH-NH_3 scan, the scan goes from maximum to minimum separation.

0
N 0.00000000 0.00000000 0.11202000
H 0.00000000 0.94195000 -0.26139000
H -0.81576000 -0.47098000 -0.26139000
H 0.81576000 -0.47098000 -0.26139000
H 0.00000000 0.00000000 3.68702000
F 0.00000000 0.00000000 4.61114000

1
N 0.00000000 0.00000000 0.11202000
H 0.00000000 0.94195000 -0.26139000
H -0.81576000 -0.47098000 -0.26139000
H 0.81576000 -0.47098000 -0.26139000
H 0.00000000 0.00000000 3.57702000
F 0.00000000 0.00000000 4.50114000

2
N 0.00000000 0.00000000 0.11202000
H 0.00000000 0.94195000 -0.26139000
H -0.81576000 -0.47098000 -0.26139000
H 0.81576000 -0.47098000 -0.26139000
H 0.00000000 0.00000000 3.46702000
F 0.00000000 0.00000000 4.39114000

3
N 0.00000000 0.00000000 0.11202000
H 0.00000000 0.94195000 -0.26139000
H -0.81576000 -0.47098000 -0.26139000
H 0.81576000 -0.47098000 -0.26139000
H 0.00000000 0.00000000 3.35702000
F 0.00000000 0.00000000 4.28114000

4
N 0.00000000 0.00000000 0.11202000
H 0.00000000 0.94195000 -0.26139000
H -0.81576000 -0.47098000 -0.26139000
H 0.81576000 -0.47098000 -0.26139000
H 0.00000000 0.00000000 3.24702000
F 0.00000000 0.00000000 4.17114000

5
N 0.00000000 0.00000000 0.11202000
H 0.00000000 0.94195000 -0.26139000
H -0.81576000 -0.47098000 -0.26139000
H 0.81576000 -0.47098000 -0.26139000
H 0.00000000 0.00000000 3.13702000
F 0.00000000 0.00000000 4.06114000

6
N 0.00000000 0.00000000 0.11202000
H 0.00000000 0.94195000 -0.26139000
H -0.81576000 -0.47098000 -0.26139000
H 0.81576000 -0.47098000 -0.26139000
H 0.00000000 0.00000000 3.02702000
F 0.00000000 0.00000000 3.95114000

7
N 0.00000000 0.00000000 0.11202000
H 0.00000000 0.94195000 -0.26139000
H -0.81576000 -0.47098000 -0.26139000
H 0.81576000 -0.47098000 -0.26139000
H 0.00000000 0.00000000 2.91702000
F 0.00000000 0.00000000 3.84114000

8
N 0.00000000 0.00000000 0.11202000
H 0.00000000 0.94195000 -0.26139000
H -0.81576000 -0.47098000 -0.26139000

H 0.81576000 -0.47098000 -0.26139000
H 0.00000000 0.00000000 2.80702000
F 0.00000000 0.00000000 3.73114000

9
N 0.00000000 0.00000000 0.11202000
H 0.00000000 0.94195000 -0.26139000
H -0.81576000 -0.47098000 -0.26139000
H 0.81576000 -0.47098000 -0.26139000
H 0.00000000 0.00000000 2.69702000
F 0.00000000 0.00000000 3.62114000

10
N 0.00000000 0.00000000 0.11202000
H 0.00000000 0.94195000 -0.26139000
H -0.81576000 -0.47098000 -0.26139000
H 0.81576000 -0.47098000 -0.26139000
H 0.00000000 0.00000000 2.58702000
F 0.00000000 0.00000000 3.51114000

11
N 0.00000000 0.00000000 0.11202000
H 0.00000000 0.94195000 -0.26139000
H -0.81576000 -0.47098000 -0.26139000
H 0.81576000 -0.47098000 -0.26139000
H 0.00000000 0.00000000 2.47702000
F 0.00000000 0.00000000 3.40114000

12
N 0.00000000 0.00000000 0.11202000
H 0.00000000 0.94195000 -0.26139000
H -0.81576000 -0.47098000 -0.26139000
H 0.81576000 -0.47098000 -0.26139000
H 0.00000000 0.00000000 2.36702000
F 0.00000000 0.00000000 3.29114000

13
N 0.00000000 0.00000000 0.11202000
H 0.00000000 0.94195000 -0.26139000
H -0.81576000 -0.47098000 -0.26139000
H 0.81576000 -0.47098000 -0.26139000
H 0.00000000 0.00000000 2.25702000
F 0.00000000 0.00000000 3.18114000

14
N 0.00000000 0.00000000 0.11202000
H 0.00000000 0.94195000 -0.26139000
H -0.81576000 -0.47098000 -0.26139000
H 0.81576000 -0.47098000 -0.26139000
H 0.00000000 0.00000000 2.14702000
F 0.00000000 0.00000000 3.07114000

15

N 0.00000000 0.00000000 0.11202000
H 0.00000000 0.94195000 -0.26139000
H -0.81576000 -0.47098000 -0.26139000
H 0.81576000 -0.47098000 -0.26139000
H 0.00000000 0.00000000 2.03702000
F 0.00000000 0.00000000 2.96114000
